# Molecular Phylogeny and Barcoding of *Caulerpa* (Bryopsidales) Based on the *tuf*A, *rbc*L, *18S rDNA* and *ITS rDNA* Genes

**DOI:** 10.1371/journal.pone.0082438

**Published:** 2013-12-05

**Authors:** Mudassar Anisoddin Kazi, C. R. K. Reddy, Bhavanath Jha

**Affiliations:** 1 Discipline of Marine Biotechnology and Ecology, CSIR- Central Salt and Marine Chemicals Research Institute, Bhavnagar, Gujarat, India; 2 Academy of Scientific and Innovative Research (AcSIR), CSIR, New Delhi, India; Chang Gung University, Taiwan

## Abstract

The biodiversity assessment of different taxa of the genus *Caulerpa* is of interest from the context of morphological plasticity, invasive potential of some species and biotechnological and pharmacological applications. The present study investigated the identification and molecular phylogeny of different species of *Caulerpa* occurring along the Indian coast inferred from *tuf*A, *rbc*L, 18S rDNA and ITS rDNA nucleotide sequences. Molecular data confirmed the identification of 10 distinct *Caulerpa* species: *C. veravalensis, C. verticillata, C. racemosa, C. microphysa, C. taxifolia, C. sertularioides, C. scalpelliformis, C. serrulata, C. peltata* and *C. mexicana*. All datasets significantly supported the sister relationship between *C. veravalensis* and *C. racemosa* var. *cylindracea*. It was also concluded from the results that the specimen identified previously as *C. microphysa* and *C*. *lentillifera* could not be considered as separate species. The molecular data revealed the presence of multiple lineages for *C. racemosa* which can be resolved into separate species. All four markers were used to ascertain their utility for DNA barcoding. The *tuf*A gene proved a better marker with monophyletic association as the main criteria for identification at the species level. The results also support the use of 18S rDNA insertion sequences to delineate the *Caulerpa* species through character-based barcoding. The ITS rDNA (5.8S-ITS2) phylogenetic analysis also served as another supporting tool. Further, more sequences from additional *Caulerpa* specimens will need to be analysed in order to support the role of these two markers (ITS rDNA and 18S insertion sequence) in identification of *Caulerpa* species. The present study revealed the phylogeny of *Caulerpa* as complete as possible using the currently available data, which is the first comprehensive report illustrating the molecular phylogeny and barcoding of the genus *Caulerpa* from Indian waters.

## Introduction

The siphonous green algal taxa, particularly those belonging to the genus *Caulerpa*, poses considerable difficulty in taxonomic identification at the species level due to the phenotypic plasticity in diagnostic characters. This can be further substantiated by the fact that out of 359 species (including forms and varieties) in the genus *Caulerpa*, only 85 are taxonomically valid [1]. The previous reports have shown that the assimilators and ramuli that are used as taxonomic keys in identification seems to be under the control of environmental factors such as temperature, irradiance, water movement, etc. [2-4]. Thus, conventional diagnostic characters alone have rather limited application when determining the correct identification and phylogeny of the species. 

The classical accounts of the species of *Caulerpa* were given by Agardh [5] and Weber-van Bosse [6] based on morphological characteristics. Subsequently, Svedelius [7] investigated the biodiversity of *Caulerpa* from Ceylon (Sri Lanka) following the work of Agardh [8]. The first taxonomically identified species of this genus from the Indian coast were *Caulerpa serrulata* (as *C. freycinetii*)*, C. lessonii* and *C. racemosa* var. *turbinata* (as *C. chemnitzia*) by De Toni [9], although subsequent studies added several species. A new species, *C. veravalensis*, containing narrow, linear, non-overlapping, flat pinnules with a rounded apex was described from Veraval, Gujarat, India [10]. Rao [11] described *C. mexicana* f. *indica* from North Andaman. Duraiswamy [8,12,13] prepared a comprehensive account of *Caulerpa* describing 21 taxa from the Indian shores based on morphological, cytological, anatomical and secondary metabolites (caulerpin, caulerpicin and β-sitosterol). However, these studies largely consisted of collections made from the southern part only. The biodiversity assessment study from the Gujarat coast reported the occurrence of 14 species including five varieties and three forms [14].

The genus *Caulerpa* has attracted attention recently because of the invasive nature of some species [15]. The secondary metabolites from the *Caulerpa* were also reported to have various biotechnological and pharmacological applications [16]. Some taxa belonging to the genus *Caulerpa* showed relatively well-defined morphological characters that can be easily differentiated. Nevertheless, validation of the reported *Caulerpa* species is necessary because of clear evidence of adaptive variation in their morphology, which has resulted in a series of varieties and forms. De Senerpont Domis et al. [17] suggested the need for detailed study of the *C. racemosa* complex, which harbours a number of varieties and forms. The recent molecular study on the *C. racemosa*-*C. peltata* complex revealed the presence of six different lineages that can be differentiated into species-level entities [18]. Therefore, correct taxonomic identification of the species from this genus is of paramount importance in biodiversity assessment studies. 

Although certain chemotaxonomic markers based on secondary metabolites have been considered as an additional tool, their utility in taxonomy is limited. The recent development of molecular-marker-based characterization in several groups of seaweeds has opened up new opportunities for studying phylogenetics and resolving the taxonomic issues of cryptic species. A wide range of molecular markers has been employed in the past to decipher the identification and phylogeny of the genus *Caulerpa* [18-30]. Phylogeny primarily reflects the evolutionary relationships among organisms. Moreover, the rate of evolution is variable for different molecular markers; therefore to resolve the phylogenetic relationship it usually requires extensive sequencing of multiple molecular markers. 

Herbert et al. [31] proposed the use of DNA barcodes, i.e. small DNA sequences amplified and sequenced from a standardized portion of the genome to identify and discriminate species. The Consortium for the Barcode of Life (CBOL) has proposed the RuBisCO large subunit (*rbc*L) and *mat*K as DNA barcodes for plants [32]. Some genus of brown (e.g. *Fucus*) and red (e.g. *Dilsea* and *Mazzaella*) algae, which have high morphological plasticity and are difficult to identify, were resolved successfully by utilizing the 5’end of the cytochrome c oxidase 1 gene (COI-5P) as a DNA barcode [33-35]. The difficulty in amplification of COI-5P [36] and the absence of *matK* from green algae (except Charophyte [37]) make them inappropriate candidates for barcoding in *Caulerpa*. Therefore, there is a need to develop an efficient DNA barcode system based on small DNA sequence amplified and sequenced from a standardized portion of the genome that is able to identify the *Caulerpa* species, thereby helping to explore its cryptic diversity. 

The relatively conserved *tuf*A gene is a preferred marker for identification and phylogeny of green algal taxa [18,23,36]. Saunders and Kucera [36] evaluated several markers for marine green macroalgae, albeit not *Caulerpa*, and proposed *tuf*A as the preferred barcode. The stability of the *rbc*L exon, with high amino acid sequence similarity, makes it another useful and reliable marker for such studies [38]. The 18S nuclear rDNA has been widely used in phylogenetic studies since it comprises highly conserved regions among the species and shows a high degree of functional constancy with a slow evolutionary rate. However, the highly conserved nature reduces the genetic distance between species in pairwise distance analysis if the complete locus is utilized [39]. However, a characteristic intronic insertion sequence is present in the *Caulerpa* 18S rDNA sequence. These insertion sequences were reported by Kooistra [40] in two Caulerpacean specimens, and were utilized by Durand et al. [24] for phylogenetic analysis. We have utilized these 18S introns for character-based barcoding in the present study. The molecular marker ITS rDNA shows high variability in its sequence as well as in its length, which can be exploited for comparing the *Caulerpa* populations at the inter- and intraspecific levels [19]. 

The use of molecular markers for identification and phylogenetic studies of the genus *Caulerpa* from India has not been reported to date. The present study thus investigates the utility of *tuf*A, *rbc*L and ITS rDNA by standard barcode methods (Neighbour-joining (NJ) analysis and nucleotide-sequence divergences) as described by Hebert et al. [31], the monophyletic association of taxa in phylogenetic trees [41] and 18S rDNA introns by character-based analysis as a DNA barcode to identify *Caulerpa* species. An extensive phylogenetic reconstruction was also accomplished to elucidate the relationships and the phylogenetic placement of *Caulerpa* species from Indian waters. Additionally, possible congruence between morphology and molecular data was also analysed in the present study.

## Materials and Methods

### Ethics Statement

The Chief Conservator of Forest, Marine National Park Jamnagar, Government of Gujarat, India permitted the collection of *Caulerpa* specimens from Poshitra Rocks and the collection from Krusadai Island was permitted by the Chief Wild Life Warden, Gulf of Mannar Biosphere Reserve, Government of Tamil Nadu, India. The other sampling locations are not the part of any national parks or protected areas and do not require any specific permits. It is further to confirm that the field studies did not involve endangered or protected species.

### Collection of samples and morphological identification

Repeated sampling was performed at 16 different locations, which seemed to cover nearly all *Caulerpa* species reported from India. The species diversity in the genus *Caulerpa* is concentrated along the northwest (Gujarat) and southeast (Tamil Nadu) coast of India [42]. Therefore, intensive sampling was performed mainly from these areas. The detailed morphological descriptions, images and references used for identification [43-56] for collected specimens are given in the supporting information (**Figures S1-S19 **in File **S1**). In total, 29 *Caulerpa* specimens (20 species including seven varieties and three forms and two unidentified taxa) were investigated based on the morphological differences for molecular barcoding and phylogenetic analysis. The collection sites for the specimens included in this study are shown in Figure **S1**. Specimens used in the analyses, and the specimen voucher numbers, collection sites and accession numbers, are listed in Table **S1**. Samples were cleaned with sterile seawater to remove mud and epiphytes and finally rinsed with distilled water. Specimens were stored at -20°C before genomic DNA isolation. Voucher specimens for individual species were submitted to the Taxonomic Reference Centre for seaweeds at the Council of Scientific and Industrial Research-Central Salt and Marine Chemicals Research Institute (CSIR-CSMCRI). 

### DNA extraction, amplification and sequencing

Genomic DNA was isolated by a modified CTAB DNA extraction method [57]. Amplification by PCR was performed in a master mix of volume 25 µL containing 5 pmol of each primer; 200 µM of each dNTP; 1X assay buffer; and 1.25 units of *Taq* DNA polymerase. The details of the molecular markers, primers and amplification conditions utilized in this study are summarized in Table **1**. Amplifications were carried out using a PCR system (Bio-Rad, Hercules, CA, USA). PCR products were purified and subjected to commercial sequencing (Macrogen Inc., Korea).

**Table 1:** Primer details and PCR conditions used for the amplification of 18S rDNA, ITS rDNA, *rbc*L and *tuf*A molecular markers used in this study.

**Primer**	**Primer Sequences (5’-3’)**	**DNA Markers**	**PCR conditions**	**Reference**
18SF 18SR	CAACCTGGTTGATCCTGCCAGT TGATCCTTCTGCAGGTTCACCTAC	18S rDNA	94°C ⁄ 5 min; 35cyclesof 94°C ⁄ 1min, 52.4°C ⁄ 1 min 72°C ⁄ 2min; final extension 72°C ⁄ 5 min	[73]
ITSF ITSR	CCTCTGAACCTTCGGGAG TTCACTCGCCATTACT	ITS rDNA	94°C ⁄ 5 min; 35 cycles of 94°C ⁄ 1 min, 52.4°C ⁄ 1 min, 72°C ⁄2 min; final extension 72°C ⁄5 min	[20]
rb-F rb-R	GCTTATGCWAAAACATTYCAAGG AATTTCTTTCCAAACTTCACAAGC	*rbc*L	94°C ⁄ 5 min; 35 cycles of 94°C ⁄ 45 sec, 41.5°C ⁄ 45 sec, 72°C ⁄2 min; final extension 72°C ⁄10 min	[27]
Tuf-F Tuf-R	TGAAACAGAAMAWCGTCATTATGC CCTTCNCGAATMGCRAAWCGC	*tuf*A	94°C ⁄ 5 min; 35 cycles of 94°C ⁄ 1 min, 50°C ⁄ 1 min, 72°C ⁄2 min; final extension 72°C ⁄5 min	[23]

IUPAC nucleotide ambiguity codes: W= A/T, Y= C/T, M= A/C, N= A/T/C/G

### Data analysis

Individual sequences obtained in this study were compared with accessions in the National Center for Biotechnology Information (NCBI) (Table **S1**) using BLAST analysis. The additional sequences were retrieved from GenBank in order to compare the inter- and intraspecific nucleotide divergences and to produce the phylogeny of *Caulerpa* as complete as possible using the currently available data. *Caulerpella ambigua* was used as an outgroup in *tuf*A and *rbc*L tree as it was found to be the most basal taxon to all the *Caulerpa* species [23,28]. Multiple sequence alignment was performed with MAFFT version 6 with Q-INS-i strategy activated (which considers secondary-structure information of RNA for alignment) for ITS rDNA and 18S rDNA insertion sequence alignment [58]. 

The estimation of nucleotide divergence between sequences was calculated using the Kimura 2- parameter (K2P) for ITS rDNA, Tamura-Nei (TN93) for *rbc*L and Tamura 3-parameter (T92) for the *tuf*A dataset. The rate variation among sites was modelled with a gamma distribution (shape parameter = 5). Model selection analysis was conducted to calculate the best-fit model of substitution by *MEGA* v.5 [59]. A neighbour-joining (NJ) tree was constructed by the bootstrap resampling method with 1,000 bootstrap replications in *MEGA* v.5 [59]. Minimum interspecific distances and maximum intraspecific distances were calculated for each species identified and named by traditional taxonomical features. 

Phylogenetic trees were constructed by Bayesian inference (BI) using MrBayes v.3.1.2 [60]. Model selection analysis was conducted to calculate the best-fit model of nucleotide evolution by jModelTest 0.1.1 [61,62]. A codon-based partition strategy was used for *rbc*L and *tuf*A gene datasets. The best scheme of model substitutions for partitioned data was generated through PartitionFinder v.1.0 [63]. The models were selected based on the Bayesian Information Criterion (BIC) [64] scores for each dataset (Table **2**).

**Table 2:** Nucleotide substitution models for respective datasets for Bayesian analysis.

**Dataset**	**No. of sequences**	**Subset Partitions**	**Model**	**BIC**	**ln*L***
*rbc*L	45	p1 = 1-1076\3 p2 = 2-1076\3 p3 = 3-1076\3	HKY+G JC HKY+G	7300.60	-3304.74
ITS rDNA	62		HKY+G	13695.80	- 6445.29
*tuf*A	82	p1 = 1-815\3 p2 = 2-815\3 p3 = 3-815\3	GTR+G F81+G GTR+I+G	9917.05	-4335.13

BIC, Bayesian Information Criterion; ln*L*, Maximum Likelihood value; +G, Gamma distribution; JC, Jukes-Cantor; GTR, General Time Reversible; HKY, Hasegawa-Kishino-Yano; F81, Felsenstein 1981; p1, p2, p3, partition of dataset based on codon position.

The Markov chain Monte Carlo (MCMC) method was used for Bayesian phylogenetic analyses. Each analysis consisted of three heated and one cold Markov chains. Sample and print frequency was set to 100 and 1,000 respectively for 2,000,000 generations. The 50 per cent majority rule consensus tree was obtained after discarding 25% of sampled trees as burn-in.

## Results

### Morphological identification

The morphological characters of collected specimens were studied by following the traditional taxonomic keys for the genus *Caulerpa*. In total, 20 species including seven varieties and three forms were identified (**Figures S1-S19 **in File **S1**). The cryptic nature of two taxa (*Caulerpa* sp. C03 and *Caulerpa* sp. C13) made it difficult to identify them at the species level. Two specimens of *C. veravalensis* (C10 & C23) resembling the specimen described by Thivy and Chauhan [10] were selected for molecular analysis. These specimens were characterized by a pinnately divided flat broad midrib, opposite to alternate flat ramuli with a rounded apex and occasional bifurcation in apices of ramuli (**Figure S9 **in File **S1**). The *C. scalpelliformis* (C21), var. *denticulata* (C12) and forma *dwarkensis* (C01) were differentiated following the treatment given by Børgesen [65]. *C. scalpelliformis* var. *denticulata* was characterized by denticulation along the outer marginal lobes (**Figure S1 **in File **S1**). The forma *dwarkensis* has an alternate arrangement of same-length ramuli throughout, except at the top, on regularly divided assimilators (**Figure S1 **in File **S1**). The morphology of the specimen C29 agrees very well with *C. racemosa* var. *racemosa* f. *remota* Coppejans described by Coppejans et al. [66] except the length of rachis, which ranged from 1.5 to 5 cm (**Figure S17 **in File **S1**). Two specimens, C05 (collected from the western coast of India) and C20 (collected from the eastern coast of India), had cylindrical, sometimes laterally compressed, ramuli that were radially arranged on assimilators (**Figure S4 **in File **S1**). There is a strong resemblance of these specimens to the description of *C. racemosa* var. *laetevirens* f. *laxa* by Børgesen [65] and with *C. racemosa* var. *cylindracea* f. *laxa* described by Coppejans et al. [66]. The specimen C06 was identified as *C. microphysa* characterized by far fewer vesicles that are not clear in longitudinal series on the axis, short assimilators up to 3.0 cm and spherical ramuli up to 2.0 mm diameter (**Figure S5 **in File **S1**). Specimen C14, however, with erect assimilators up to 10 cm long, and densely covered with spherical to sub-spherical ramuli and ramuli with constricted pedicels, was identified as *C. lentillifera* (**Figure S11 **in File **S1**).

### Barcoding Analysis

**Figure 1:**
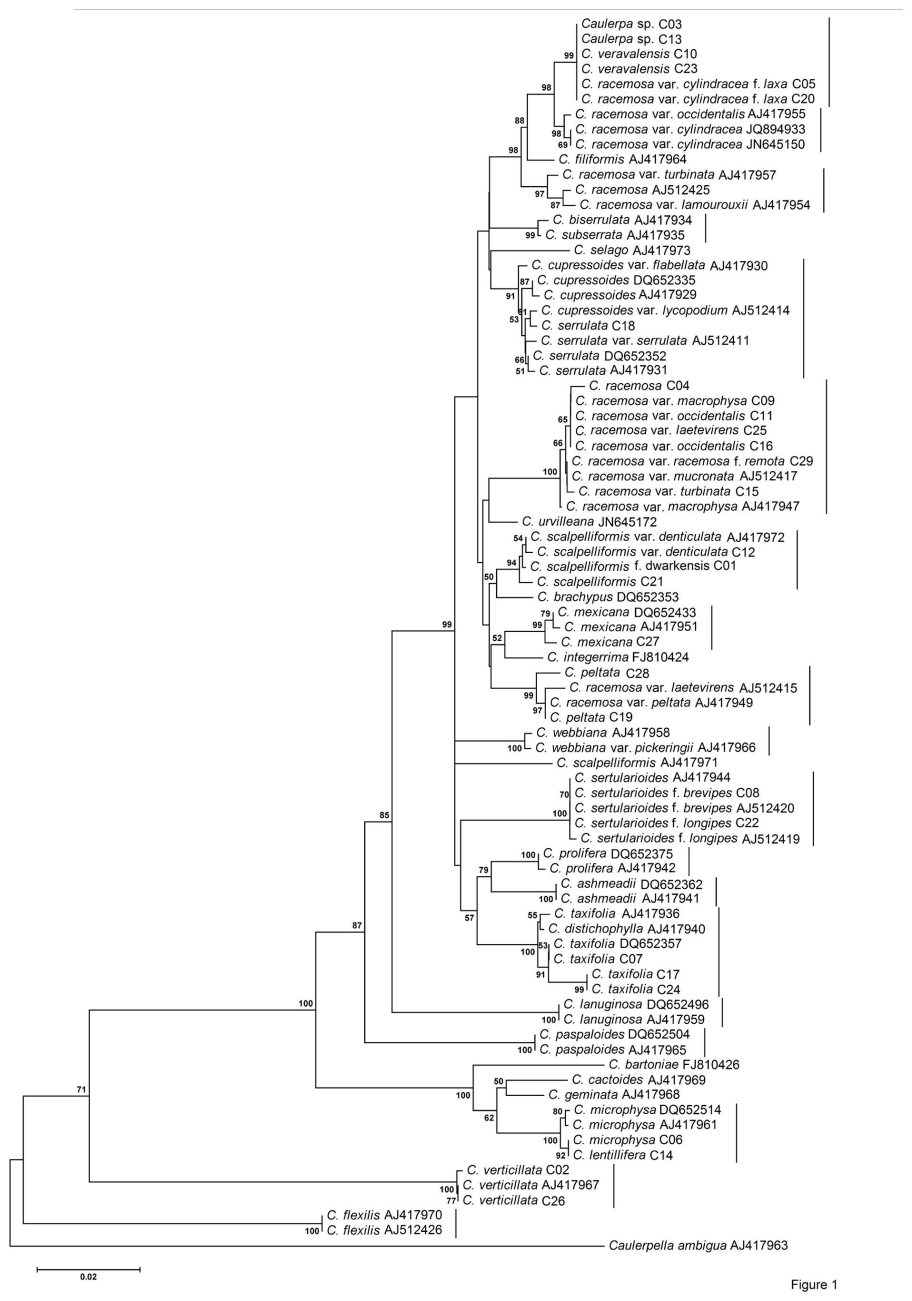
NJ tree based on *tuf*A gene sequence data. Support values at nodes correspond to bootstrap proportion (BS). Sample ID for specimens from this study and accession numbers for the reference sequences are given for identification in Table S1. Solid lines on the right indicate possible clades.

**Figure 2:**
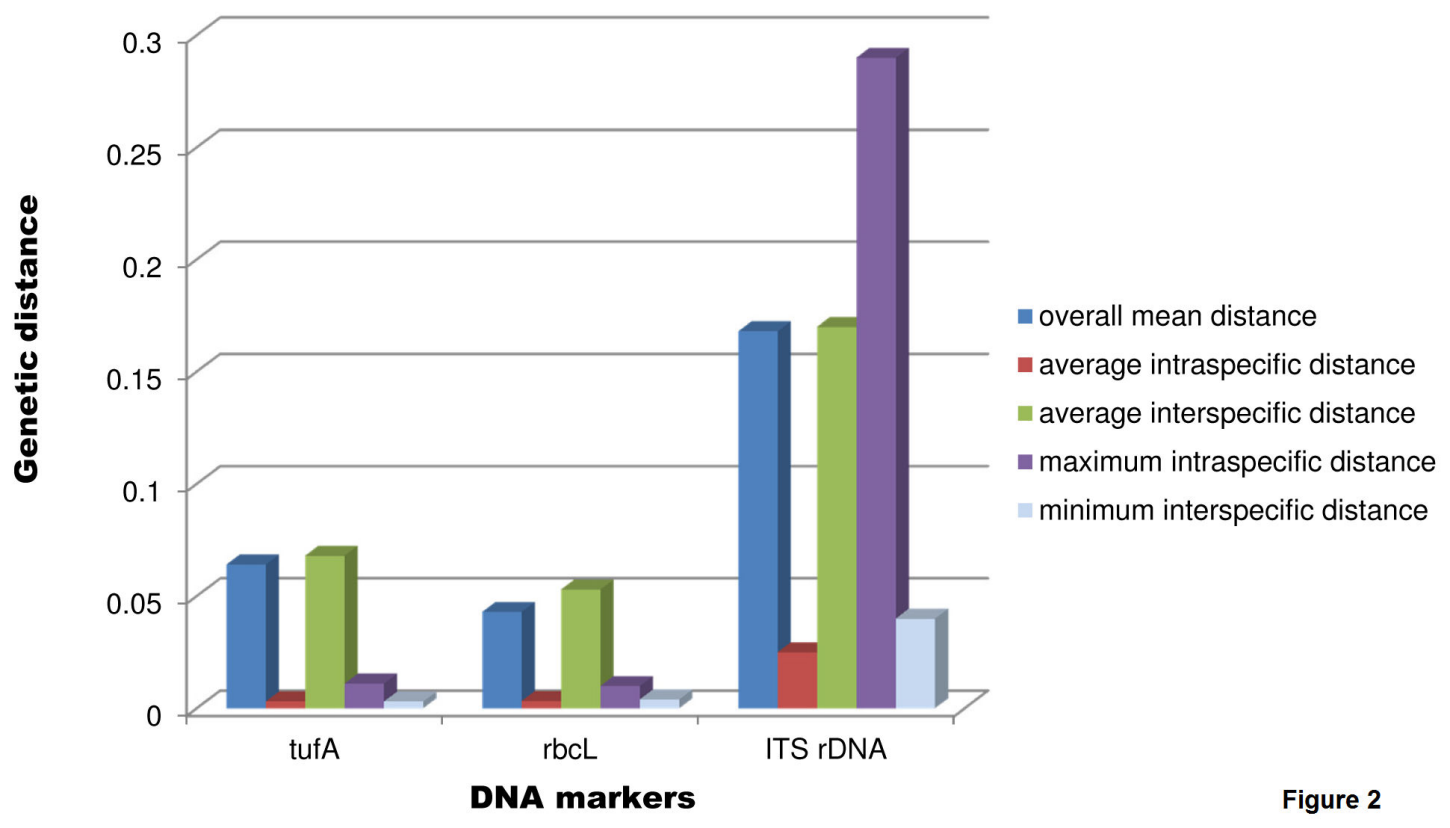
Plot of intra- and interspecific genetic distances for the *tuf*A, *rbc*L and ITS rDNA molecular markers.

A total of 82 *tuf*A sequences were aligned to generate the dataset of total length of 815 nucleotide positions in alignment to construct the NJ tree and to calculate pairwise distance. The NJ tree (Figure **1**) revealed the presence of 19 distinct well-supported clades. The average corrected divergence over all sequence pairs was 0.063. The average intraspecific genetic divergence was 0.003 and the average interspecific divergence was 0.068. The divergence between morphologically different species *C. serrulata* and *C. cupressoides* was exceptionally low (from 0.003 to 0.004). The morphologically distinct species *C. subserrata* (AJ417935) and *C. biserrulata* (AJ417934) showed interspecific variation of 0.003. The pairwise distance analysis of *tuf*A gene data showed the intraspecific variation ranged from 0.0 to 0.011 whereas interspecific variation ranged from 0.003 to 0.173. The result clearly indicates the overlapping of maximum intraspecific and minimum interspecific genetic distances (Figure **2**). The highest divergence was observed for the *C. verticillata* with a mean genetic distance 0.160 (0.147-0.173). The specimen identified previously as *C. microphysa* and *C. lentillifera* showed no sequence divergence for all the markers studied. In view of this, these two taxa were excluded from the interspecific variation range.

A total of 45 sequences of the *rbc*L gene were used to generate the dataset of total length of 1076 nucleotide positions in alignment. The intron was removed from the *rbc*L gene sequences before analysis. In total, 10 clusters were recovered in NJ analysis (Figure **S2**). The cluster with *C. mexicana* (C27) and *C. cuppressoides* var. *lycopodium* (AJ512470) was poorly supported among these clusters. The average divergence over all sequence pairs was 0.043. The average intraspecific genetic divergence was 0.0033 and the average interspecific divergence was 0.053. The maximum intraspecific genetic distance (0.010) exceeds the minimum interspecific distance (0.004) in the present dataset. *C. verticillata* showed the highest divergence with a mean genetic distance of 0.127 (0.121-0.132). 

In addition to the above markers, we sequenced the ITS1-5.8-ITS2 region of the rDNA. We tried to align 62 ITS rDNA sequences, but the ITS1 region was virtually impossible to align and was removed before further analysis. In NJ analysis, 13 distinct clusters were recovered with high support values (Figure **S3**). All species studied were clearly differentiated into distinct clades. The average divergence over all sequence pairs was 0.168. The average intraspecific genetic divergence was 0.025 and the average interspecific divergence was 0.17. The nucleotide divergence varied from 0.003 to 0.301 within species whereas interspecific variation ranged from 0.04 to 0.661. The highest divergence was observed for the *C. verticillata* with a mean genetic distance 0.510 (0.469-0.661).

**Figure 3:**
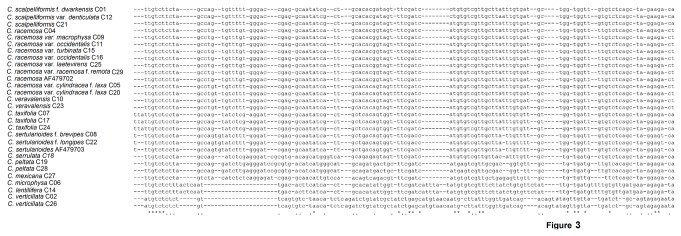
Bayesian phylogenetic tree based on *tuf*A gene sequence data. Support values at nodes correspond to posterior probabilities (pp). Sample ID for specimens from this study and accession numbers for the reference sequences are given for identification in Table S1, Solid lines on the right indicate possible clades.

The length of insertion sequence in 18S rDNA sequence was found to be in the range of 113-115 nucleotides in *Caulerpa* species. The insertion and deletion pattern in insertion sequence was species specific and could be utilized for species-level identification by a character-based approach. The alignment of 18S rDNA insertion sequences (Figure **3**) clearly differentiated the taxa at the species level. *C. microphysa* (C06) and *C. lentillifera* (C14) shared an identical insertion sequence. *C. racemosa* var. *cylindracea* f. *laxa* and *C. veravalensis* were separated by a single diagnostic character.

### Phylogenetic analysis

**Figure 4:**
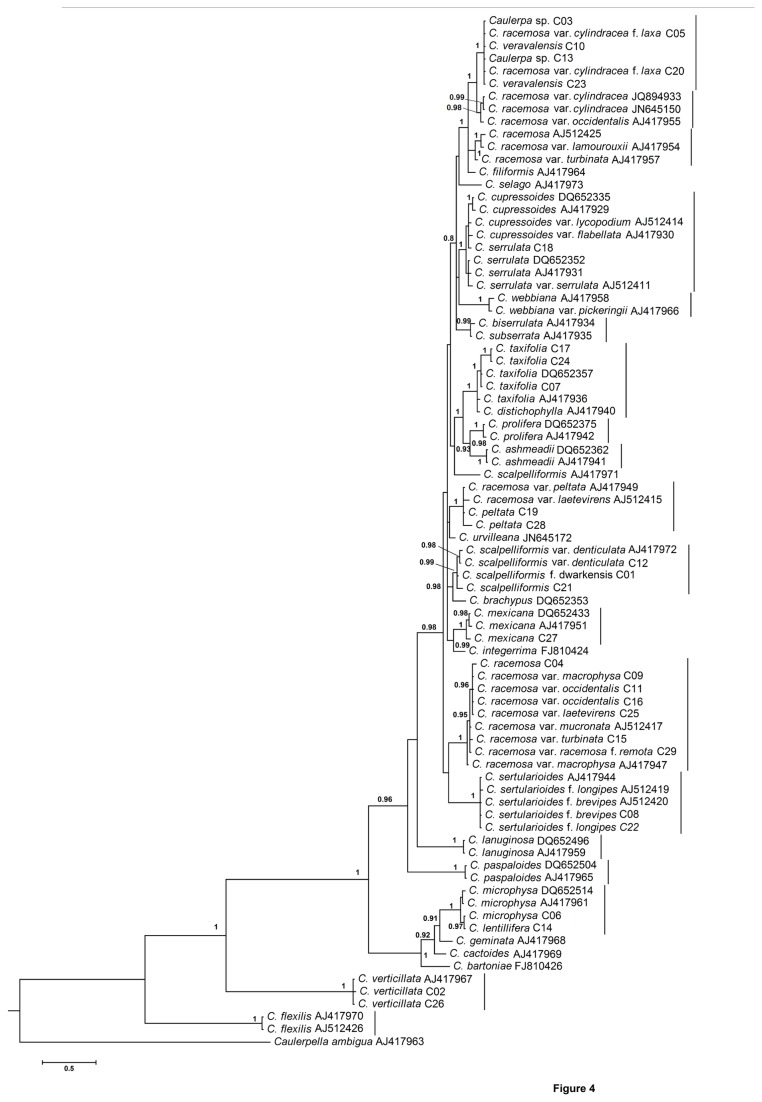
Sequence alignment of 18S rDNA insertion sequences for listed *Caulerpa* species revealed specific insertion-deletion pattern.

The Bayesian phylogenetic tree of the *tuf*A gene (Figure **4**) supported the differentiation of species depicted in the NJ tree. It was observed that *C. racemosa* var. *cylindracea* f. *laxa* (C05 and C20) clustered with *C. veravalensis* with strong support (posterior probability (pp) =1.0). This clade was a sister lineage to *C. racemosa* var. *cylindracea* (pp=1.0). Similarly, *C. racemosa* var. *peltata* (AJ417949) and *C. racemosa* var. *laetevirens* (AJ512415) clustered with *C. peltata* (C19 and C28) with very strong support (pp=1.0). No sequence difference was observed for *C. microphysa* (C06) and *C. lentillifera* (C14), which clustered together. Furthermore, *Caulerpa* sp. (C03, C13) showed no sequence difference with *C. veravalensis*, and these clustered together. The position of *C. serrulata* (C18) was clearly paraphyletic in the BI tree.

**Figure 5:**
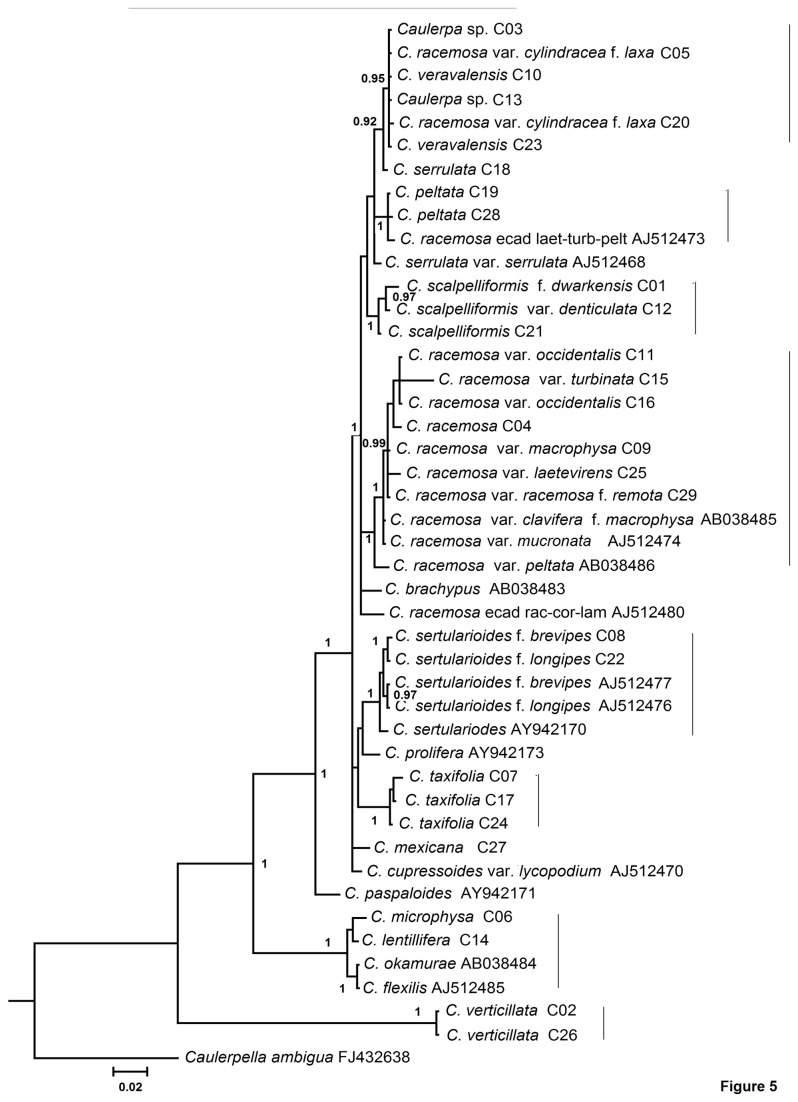
Bayesian phylogenetic tree based on *rbc*L gene sequence data. Support values at nodes correspond to posterior probabilities (pp). Sample ID for specimens from this study and accession numbers for the reference sequences are given for identification in Table S1. Solid lines on the right indicate possible clades.

The *rbc*L gene phylogeny of *Caulerpa* based on Bayesian analysis depicts the presence of eight well-supported clades with eight separate lineages (Figure **5**). *C. racemosa* var. *cylindracea* f. *laxa* (C05 and C20), *C. veravalensis* (C10 and C23) and *Caulerpa* sp. (C03, C13) clustered together with high support values (pp=0.95). *C. microphysa* (C06) and *C. lentillifera* (C14) showed no sequence difference and clustered together. Similarly no sequence difference was observed in *C. flexilis* (AJ512485) and *C. okamurae* (AB038484). The *C. racemosa* and varieties were positioned in four different lineages.

**Figure 6:**
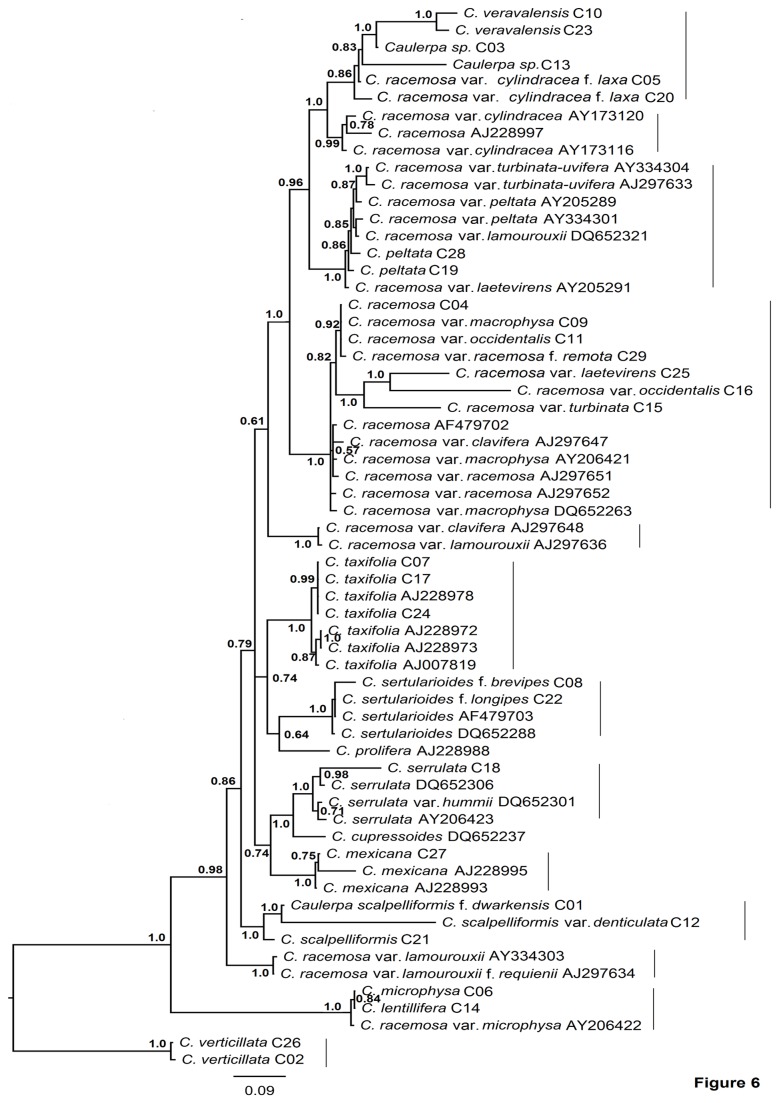
Bayesian phylogenetic tree based on ITS rDNA sequence data. Support values at nodes correspond to posterior probabilities (pp). Sample ID for specimens from this study and accession numbers for the reference sequences are given for identification in Table S1. Solid lines on the right indicate possible clades.

The Bayesian analysis of ITS rDNA resulted in a phylogenetic tree (Figure **6**) consisting of 12 well-supported and one weakly supported clade. Among these clades, six showed the presence of taxa belonging to the *C. racemosa* complex underlining the polyphyly of the complex. *C. serrulata* and *C. cupressoides* were recovered as sister lineages with strong support (pp=1.0). *C. peltata* (C19 and C28) formed a separate lineage with the species that were mostly characterized by turbinate, trumpet or peltate ramuli (pp=1.0). 

## Discussion

This study aimed to determine the identification and phylogenetics of the Indian *Caulerpa* species by employing multiple markers. This inclusive multi-gene approach has further improved the reconstruction of the phylogenetic relationship among *Caulerpa* species.

### Barcoding Analysis

In this study, the overlap between levels of intraspecific genetic distance and interspecific genetic distance was observed for all the datasets (Figure **2**). The lower interspecific genetic distance was observed in some of the taxonomically well-established species. Therefore, it is difficult to define species boundaries using a distance-based approach in *Caulerpa*. Another approach of delineation of species is through monophyletic association of taxa in a Neighbour-Joining (NJ) tree, which does not depend on the distance-based threshold method [41]. In NJ analysis of *tuf*A and ITS rDNA, most of the clades were recovered as being monophyletic with strong support with few exceptions. The Bayesian phylogenetic tree (Figure **4** and Figure **6**) supported the differentiation of species, which was also depicted in NJ trees. ITS-rDNA-based phylogenetic analysis was found to be mostly congruent with the *tuf*A gene analysis. In the *rbc*L-gene-based analysis, the position of certain taxa did not resolve sufficiently and also showed incongruence with other datasets. The *rbc*L gene was also found to be least variable in comparison with *tuf*A and ITS rDNA (Figure **2**). Handeler et al. [67] and Saunders and Kucera [36] supported *tuf*A gene as a barcode for green algae. Therefore, monophyletic association of taxa in the *tuf*A-gene-based tree can be utilized for species identification. The character-based identification of the species of *Caulerpa* by using the 18S rDNA insertion sequence was another useful identification tool that we found in this study. For example, *C. peltata* (C19 and C28) was clearly separated from *C. racemosa* and varieties in the 18S rDNA insertion sequence alignment (Figure **3**). The ITS rDNA (5.8S-ITS2) analysis can be used as an additional supporting tool for identification purpose, but more sequences from species of *Caulerpa* will need to be analysed before defining the role of ITS rDNA in species identification. For example, *C. serrulata* and *C. cupressoides* showed paraphyly in *tuf*A analysis but formed a sister lineage in ITS rDNA analysis. However, there is a single valid ITS rDNA sequence available for *C. cupressoides* in GenBank dataset and more sequences will be required for differentiating the *C. cupressioides* and *C. serrulata* as monophyletic lineages. Similarly, more sequences from additional species of *Caulerpa* will need to be analysed in order to support the role of 18S insertion sequence in identification of *Caulerpa* species. Following this molecular barcoding scheme, the identity of 10 distinct species of *Caulerpa* was confirmed from Indian waters.

### Phylogenetic analysis

The *tuf*A-gene-based phylogenetic tree (Figure **4**) was found to be congruent with those of the findings of Sauvage et al. [18], Fama et al. [23] and Stam et al. [28]. The major addition to these phylogenetic analyses was *C. veravalensis*, which was recovered as a sister lineage to *C. racemosa* var. *cylindracea. C. veravalensis* was considered as a form of *C. taxifolia* and was later differentiated as a separate species based on morphological characters [68]. The species that was identified as *C. racemosa* var. *cylindracea* f. *laxa*, based on morphology, and two unidentified *Caulerpa* sp. C03 and C13, consistently placed in the same clade with *C. veravalensis* in all the phylogenetic trees. These taxa may be considered as part of a new *C. veravalensis* complex and need further detailed investigations.

The *rbc*L phylogeny (Figure **5**) was not consistent with the phylogeny of other datasets. For example, the incongruity was observed in the position of *C. flexilis* as it formed a separate lineage in *tuf*A gene analysis and clustered with *C. okamurae, C. microphysa* and *C. lentillifera* in the *rbc*L gene phylogenetic tree. The position of *C. serrulata* also differed in *rbc*L phylogeny in comparison to *tuf*A and ITS rDNA phylogenetic analyses. In *rbc*L tree (Figure **5**) *C. serrulata* was polyphyletic whereas, it was paraphyletic in *tuf*A analysis (Figure **4**) and formed sister lineage with *C. cupressoides* in ITS rDNA analysis (Figure **6**). Similarly, De Senerpont Domis et al. [17] showed the topological differences between phylogenies inferred from *tuf*A and *rbc*L genes for the genus *Caulerpa*. De Senerpont Domis et al. [17] and Fama et al. [23] indicated the possible reasons of incongruence between these two genes such as hybridization, incomplete lineage sorting, and horizontal gene transfer (organismal- level cause) and rate heterogeneity, selection, and base/codon composition biases (genetic-level causes). Fama et al. [22] reported the high levels of intra- and inter-individual polymorphism in the rDNA ITS1 region which can affect the phylogenetic reconstruction. The removal of the ITS1 region from the ITS rDNA dataset and the use of 5.8S-ITS2 in the phylogenetic analysis resulted in a robust and well-resolved phylogenetic tree (Figure **6**).

The polyphyly of the *C. racemosa* complex was evident in the analysis of all the datasets. Sauvage et al. [18] also reported the presence of six different lineages in the *C. racemosa-peltata* complex. Most of these lineages can be resolved into separate species. For example, phylogenetic analysis strongly favoured the separate species position of *C. peltata* (C19 and C28) having distinctly peltate ramuli, which was evident from the distant placement of taxa from other *C. racemosa* clades. Furthermore, the results confirmed that *C. racemosa* var. *laetevirens* (C25) is a part of the *C. racemosa* complex and is not a separate species *C. laetevirens* Montagne. 

The results of phylogenetic analysis were consistent with the study of Yeh and Chen [26], as *C. microphysa* deviated from other species proving its taxonomic distinction. Coppejans and Beeckman [69] considered *C. lentillifera* and *C. microphysa* as separate species. From the phylogenetic trees, it was inferred that both these taxa grouped together into a single clade. Furthermore, these two taxa showed no difference in *rbc*L gene sequence. These two taxa also shared a common 18S rDNA insertion sequence. Therefore, it can be considered that these two morphologically different specimens collected from India belong to the same species. The present results also agree with the study of Olsen et al. [21] wherein the conspecific nature of *C. taxifolia* and *C. mexicana* were rejected. These species have formed separately placed clades in the phylogenetic trees. 

### Morphology vs. molecular analysis

Weber van Bosse [6] classified *Caulerpa* species into 12 sections on the basis of morphology. Similarly, Calvert et al. [70] studied the phylogeny of these 12 sections based on chloroplast ultrastructure. Duraiswamy [8] categorized the Indian *Caulerpa* species into five sections. Following this work, the taxa investigated in this study can be grouped into three sections, i.e. Filicoideae, Sedoideae and Charoideae. In the present study, it was observed that clades formed in the phylogenetic trees do not entirely follow the sectional scheme. Similar findings have been reported by Fama et al. [23] for *tuf*A-gene-based phylogenetic analysis in *Caulerpa*.

Stam et al. [28] reported the polyphyletic nature of *C. scalpelliformis*. In the present *tuf*A analysis, all *C. scalpelliformis* specimens from India (C01, C12 and C21) clustered with *C. scalpelliformis* var. *denticulata* (AJ417972) from Lebanon but away from the Australian specimen (AJ417971). On the other hand, morphologically different species *C. racemosa* var. *cylindracea* f. *laxa* (C05 and C20) and *C. veravalensis* (C10 and C23) clustered together. Thus, there was no consistent pattern observed in the relationship between morphological characters and placement in the phylogenetic tree of taxa based on the molecular markers investigated. Similarly, *C. serrulta* and *C. cupressoides* are clearly differentiable on morphological characteristics but formed paraphyletic lineages in *tuf*A analysis. Therefore, for truly understanding the placement in phylogenetic tree and proper identification of different populations of *C. scalpelliformis, C. serrulta* and *C. cupressoides* more detailed study with other molecular markers will be required. The weak supports at internal nodes in clades restrict further distinction at a lower taxonomic level. However, these sub-specific ranks can be delineated by using longer nucleotide sequences [71] or detailed transcriptome analyses [72].

## Conclusion

In the present study, we present a comprehensive phylogeny of *Caulerpa* using most of the currently available data. The findings of this study showed the phylogenetic position of the Indian *Caulerpa* population vis-à-vis other parts of the world. The study supports the use of the *tuf*A gene as a preferred marker with the monophyletic association of taxa as the main criteria for identification at the species level. The ITS rDNA (5.8S-ITS2) analysis could also be used as an additional supporting tool for identification purposes, coupled with character-based identification by 18S rDNA insertion sequences at the species level. Our molecular analyses eventually led to establishment of 10 distinct *Caulerpa* species from Indian waters. Further, the taxa identified as *C*. *racemosa* var. *cylindracea* f. *laxa* and two unidentified taxa showed close proximity with *C. veravalensis* at a molecular level despite the distinct morphological variations indicating the presence of a new *C. veravalensis* complex, which needs further detailed investigation. 

## Supporting Information

File S1 **Includes Figures S1-S19.** Morphological description and images of *Caulerpa* specimens collected for this study.
(PDF)

Figure S1 **Sample collection sites from India.**
(PDF)

Figure S2 **NJ tree based on *rbc*L gene sequence data.** Support values at nodes correspond to bootstrap proportion (BS). Sample ID for specimens from this study and accession numbers for the reference sequences are given for identification in Table S1. Solid lines on the right indicate possible clades.
(PDF)

Figure S3 **NJ tree based on ITS rDNA gene sequence data.** Support values at nodes correspond to bootstrap proportion (BS). Sample ID for specimens from this study and accession numbers for the reference sequences are given for identification in Table S1. Solid lines on the right indicate possible clades.
(PDF)

Table S1 **Specimens used in analyses, specimen voucher number, collection locations, collection date and accession numbers.**
(PDF)
