# Supplementary material for: Molecular Phylogeny and Barcoding of Caulerpa (Bryopsidales) Based on the tufA, rbcL, 18S rDNA and ITS rDNA Genes
Source: PLoS One. 2013 Dec 5;8(12):e82438. doi: 10.1371/journal.pone.0082438 (PMC3855484; doi:10.1371/journal.pone.0082438)
Supplement: File S1 — Includes Figures S1-S19. Morphological description and images of Caulerpa specimens collected for this study. (PDF) [file pone.0082438.s001.pdf]

***Caulerpa scalpelliformis* (Brown ex Turner) C. Agardh**

References: [3]: fig. 10. [6]: pl. XXIII, figs. 2,3,4. [12]: fig. 2 & 5. [14]: pp 46 & 47. [44]: pl. 4, figs. 31-37; pl. 7, figs. 70 & 71; pl. 11, fig. 87. [65]: pl. 1, fig. 1. [43]: pl. XLVI).

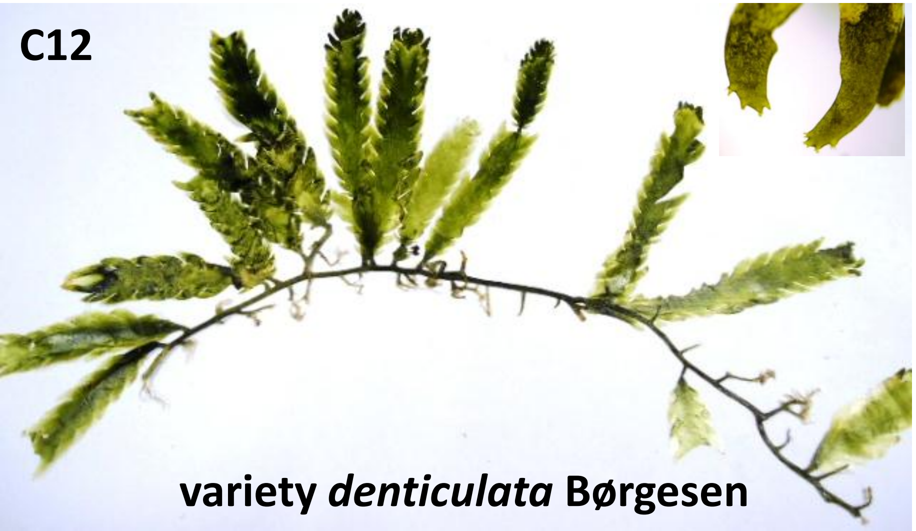

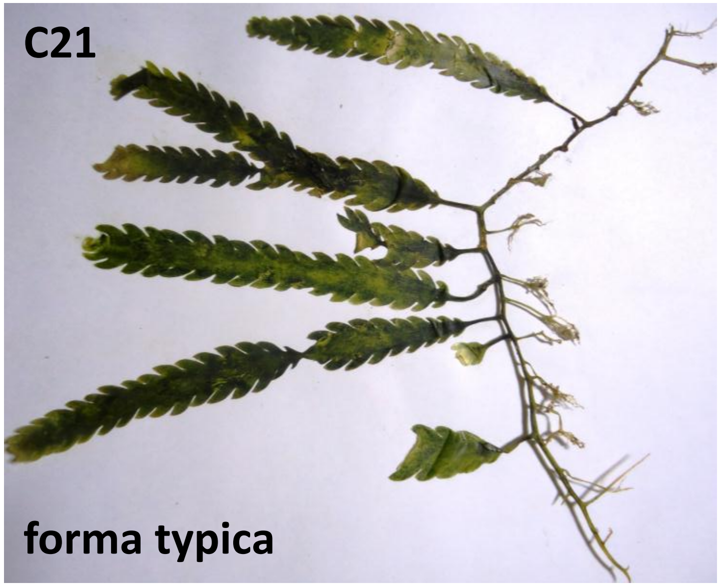

Description: Foliar assimilators upto 20 cm long, occasionally branched, upto 2-3 cm broad, flat central axis lanceolated, bilateral with vertical striations; variety *denticulata* Børgesen characterized by denticulation along the outer marginal lobes, alternate arrangement of same length ramuli throughout except at the top on regularly divided assimilators were found in forma *dwarkensis* Børgesen.

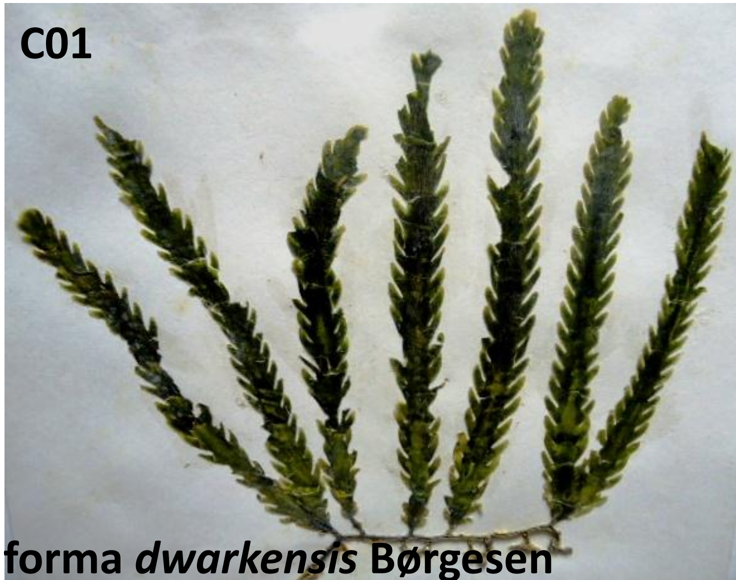

Figure S1; Specimens C01, C12, C21

Caulerpa verticillata **J. Agardh**

References : [6]: pl. XX, fig. 7. [12]: figs. 2 & 11B. [14]: p. 51. [45]: pl. 1. [66]: fig. 94.

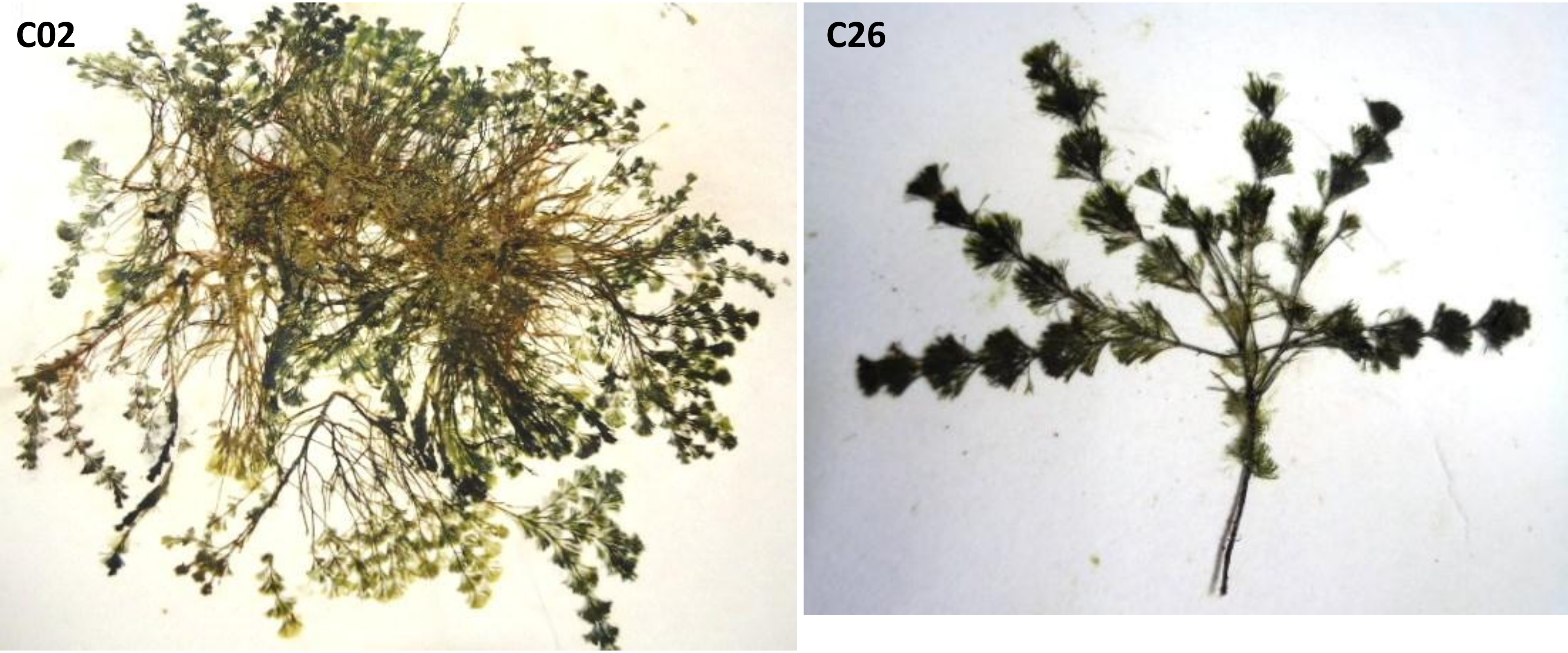

Description: Specimen completely agree with forma typica described by Thivy & Visalakshmi (1962), dark green in appearance with creeping and branched stolons; erect assimilator axes with 2-7 verticils of cylindrical determinate branchlets.

Figure S2; Specimens C02, C26

Caulerpa racemosa **(Forsskål) J. Agardh**

References: [3]: figs. 3 & 3a. [13]: figs. 6, A-H & 9 A. [14]: p 41.

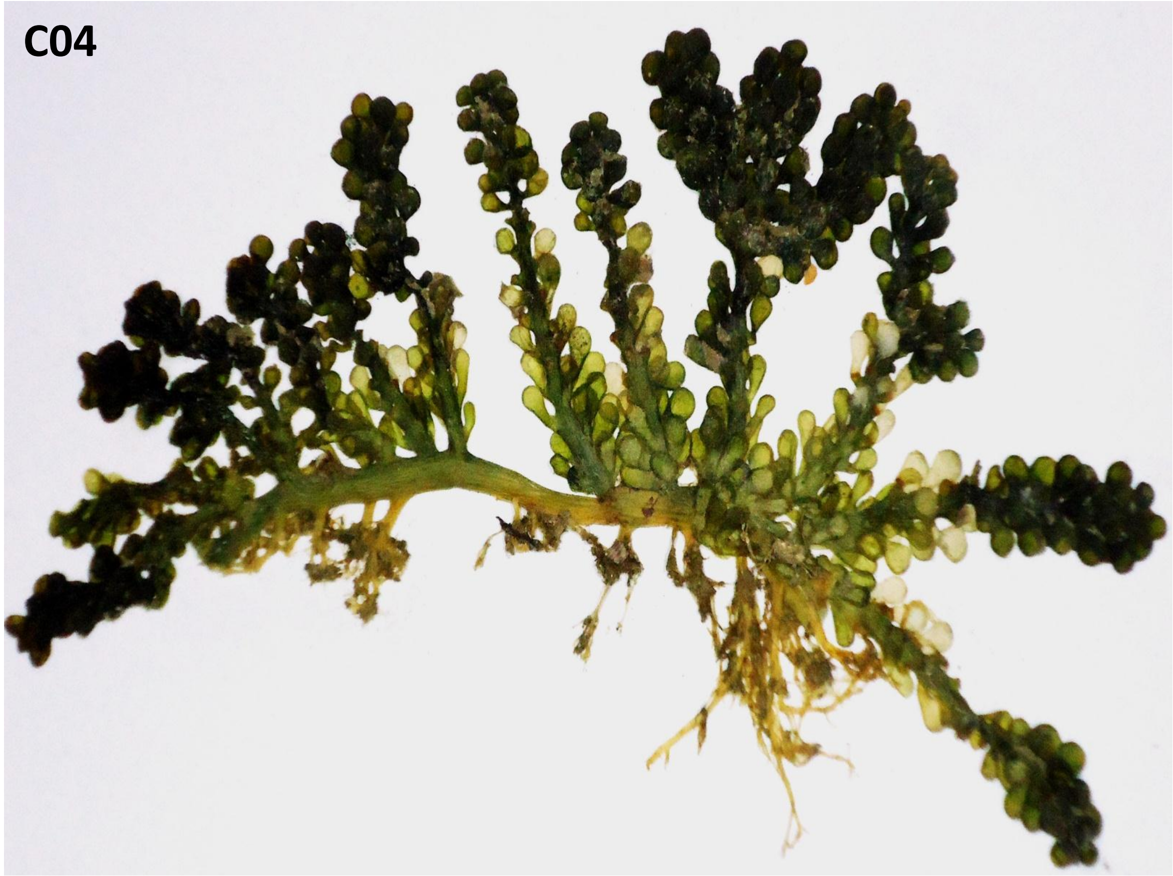

Description: Cylindrical rhizome bearing branched rhizoids; erect assimilators, cylindrical; ramuli radial, cylindrical, sub opposite and distichous.

Figure S3; Specimen C04

Caulerpa racemosa **var.** *cylindracea* **(Sonder) Verlaque, Huisman & Boudouresque f.** *laxa* **(Greville) Weber-van Bosse**

References: [65]: fig. 2 as *C. racemosa* var. *laetevirens* f. *laxa*. [66]: fig. 90.

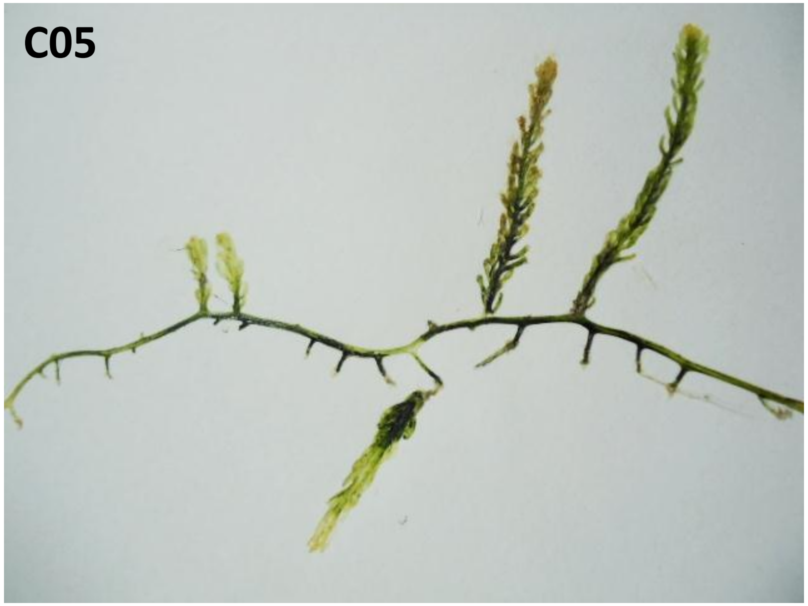

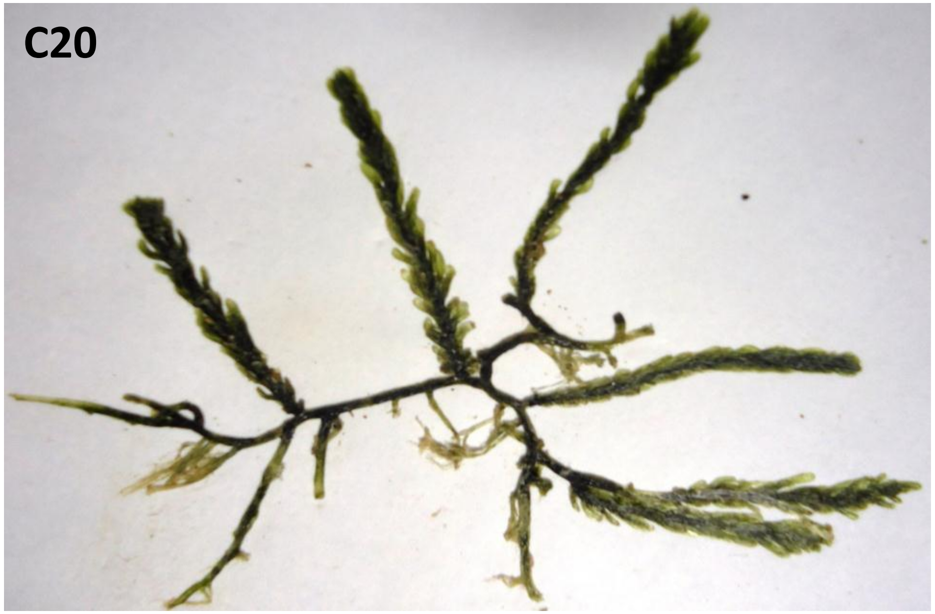

Description: Moderate size plant with few assimilators separated by large distance; ramuli cylindrical, sometimes laterally compressed, radially arranged on assimilators

Figure S4; Specimens C05, C20

Caulerpa microphysa **(Weber van Bosse) J. Feldmann**

References: [14]: p. 39. [46]: pl. 17, fig. 5; pl. 18, fig. 6. [47]: fig. 15 & 17.

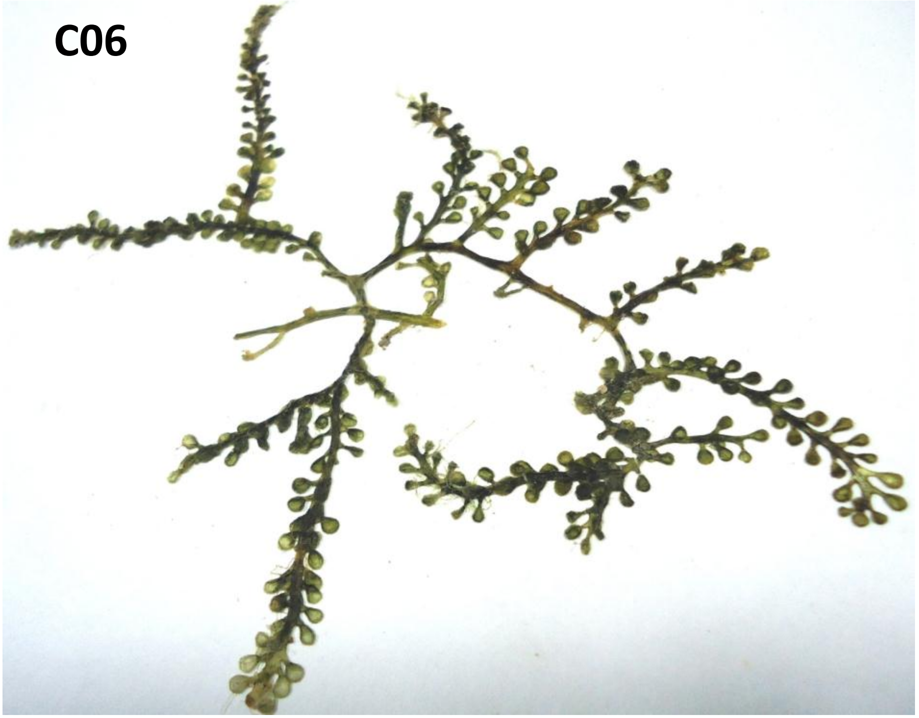

Description: Much smaller plant with far fewer vesicles which are not clearly in longitudinal series on the axis, delicate creeping; assimilators erect upto 3 cm in length and 4-6 mm in breadth; spherical ramuli upto 2.0 mm diameter.

Figure S5; Specimen C06

Caulerpa taxifolia (Vahl) C. Agardh

References : [3]: pl. VI, figs. 11 & 11a. [12]: fig. 3, A-O. [14]; p. 49. [44]: pl. 5, fig. 49; pl. 7, figs. 68 & 69; pl. 11, fig. 88. [46]: pl. 13, fig. 1. [48]: figs. 36-39. [66]: fig. 93.

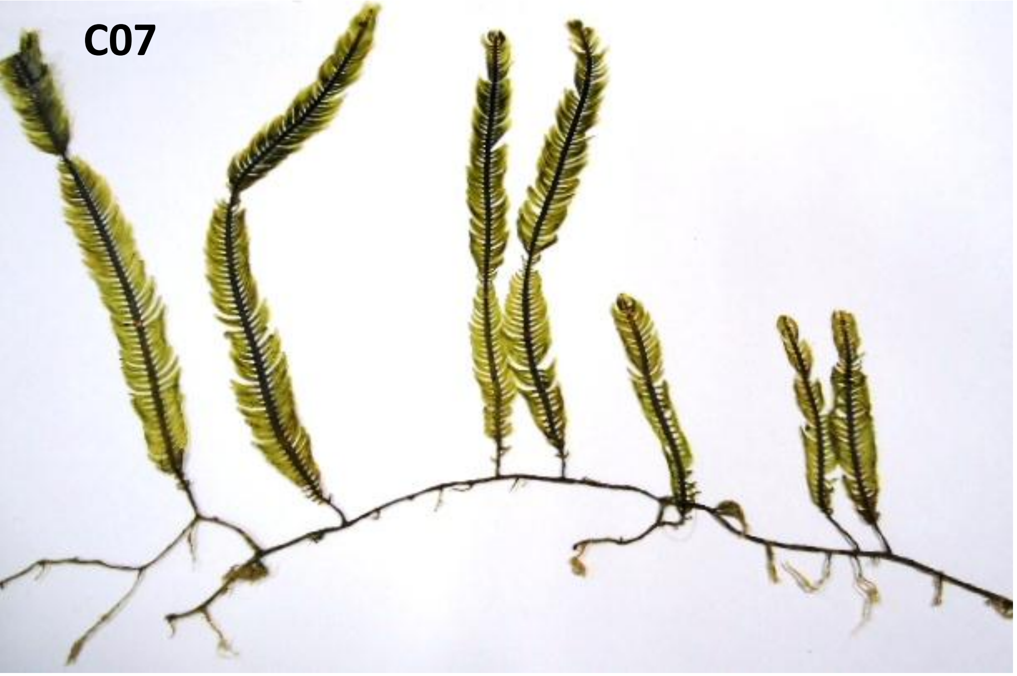

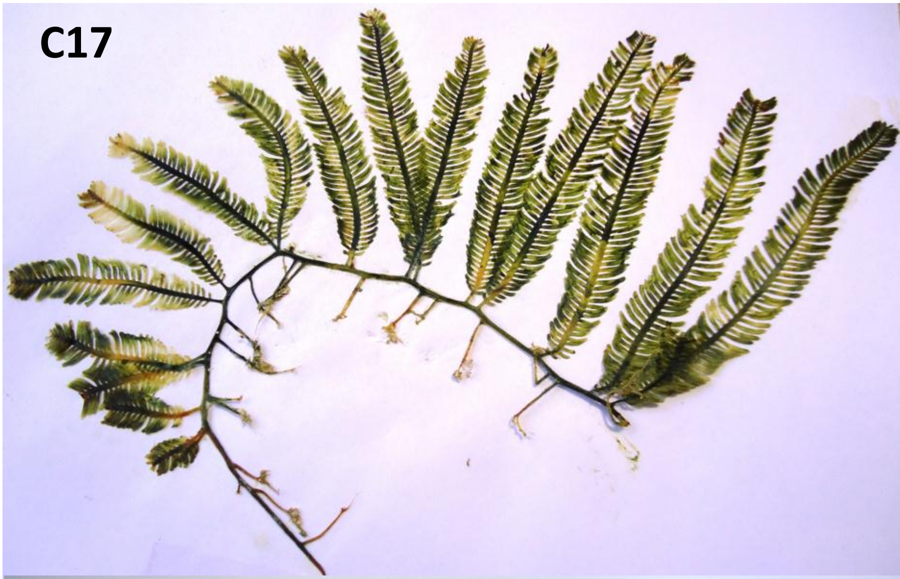

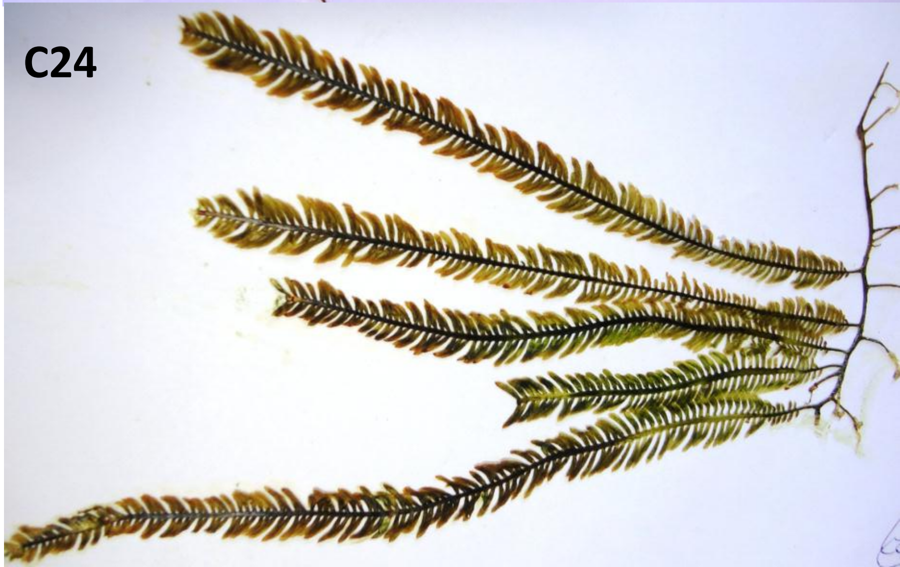

Description : Stolons creeping; assimilators erect, simple or branched, stalked, lanceolate upto 15 cm long, 8.8-13 mm broad; pinnules compressed laterally, opposite, pointed apices or mucronate, sickle shaped, constricted at base.

Figure S6; Specimens C07, C17, C24

Caulerpa sertularoides **(S. Gmelin) Howe f.** ***brevipes*** **(J. Agardh) Svedelius and f.** ***longipes*** **(J. Agardh) Collins**

References: [12]: fig. 8, A-P. [14]: p 48. [44]: pl. 7, figs. 63-67. [49]: p. 415.

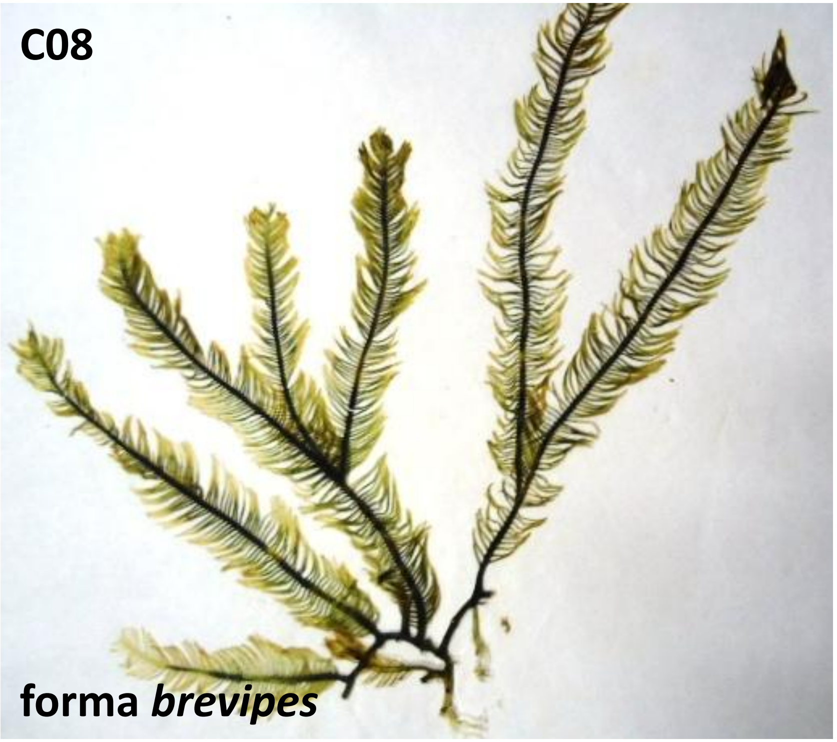

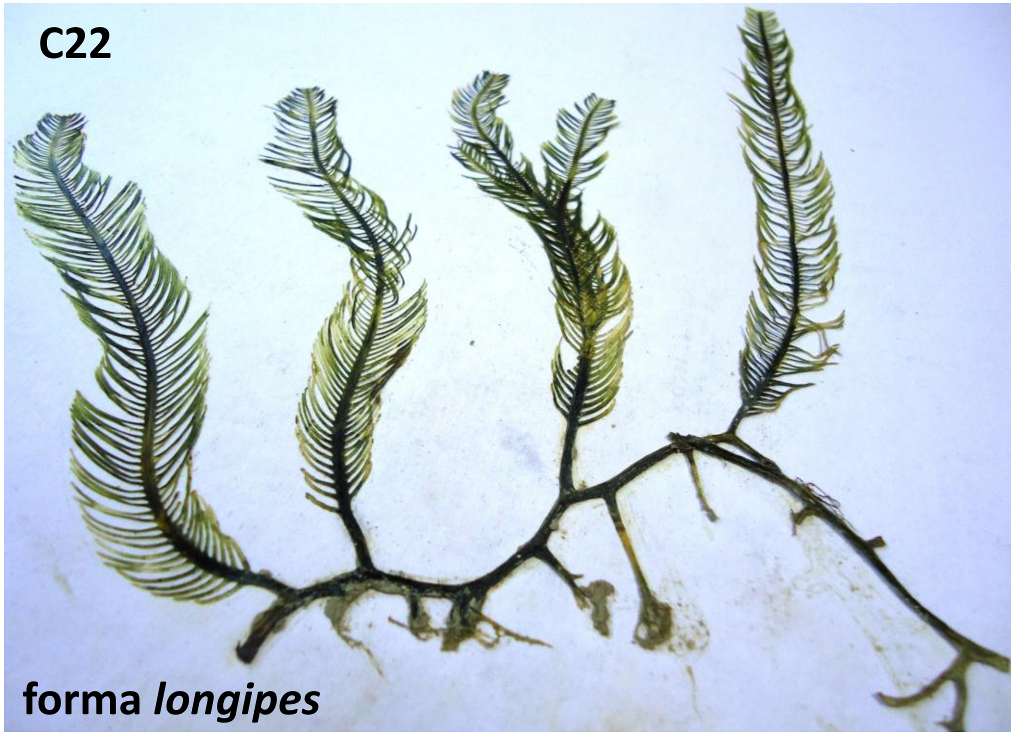

Description: Delicate plant; assimilators erect , flat, feathery in appearance; base of assimilator axis devoid of pinnules in forma *longipes* but present at the base in forma *brevipes;* pinnules opposite with pointed apices.

Figure S7; Specimens C08, C22

Caulerpa racemosa **(Forsskål) J. Agardh var.** *macrophysa* **(Sonder ex Kützing) Taylor**

References: [14]: p. 43. [62]: pl. 17, fig. 1; pl. 18, fig. 2. [66]: fig. 88, as var. *racemosa* f. *macrophysa*.

C09

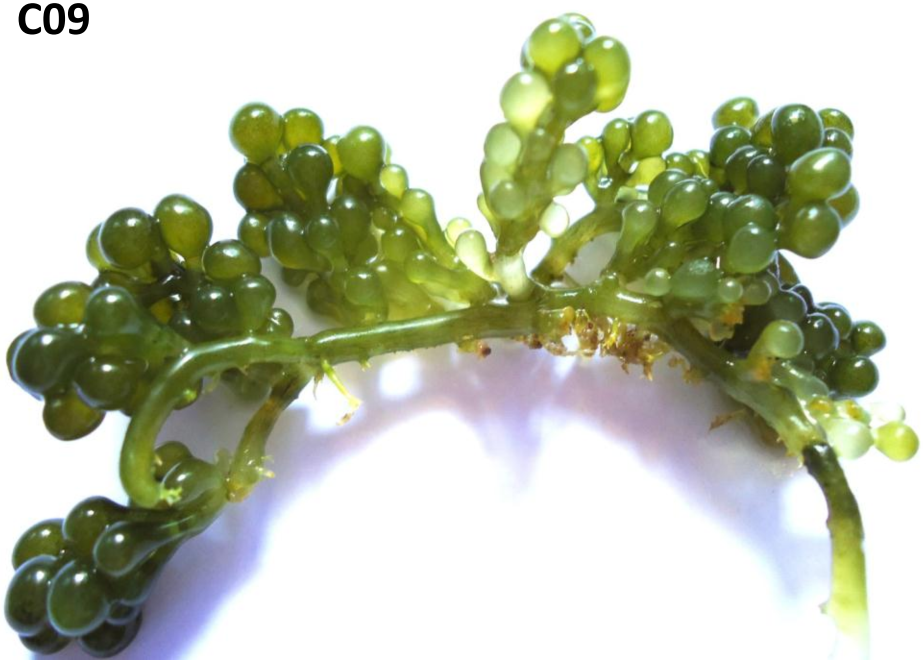

Description: Erect assimilators upto 6 cm tall; ramuli broad, hemispherical to convex, 3-5 mm in diameter.

Figure S8; Specimen C09

Caulerpa veravalensis **Thivy & Chauhan**

References: [10]: pl. 1, figs. 1, 2, & 4. [14]: p. 50. [50]: figs. 2-4.

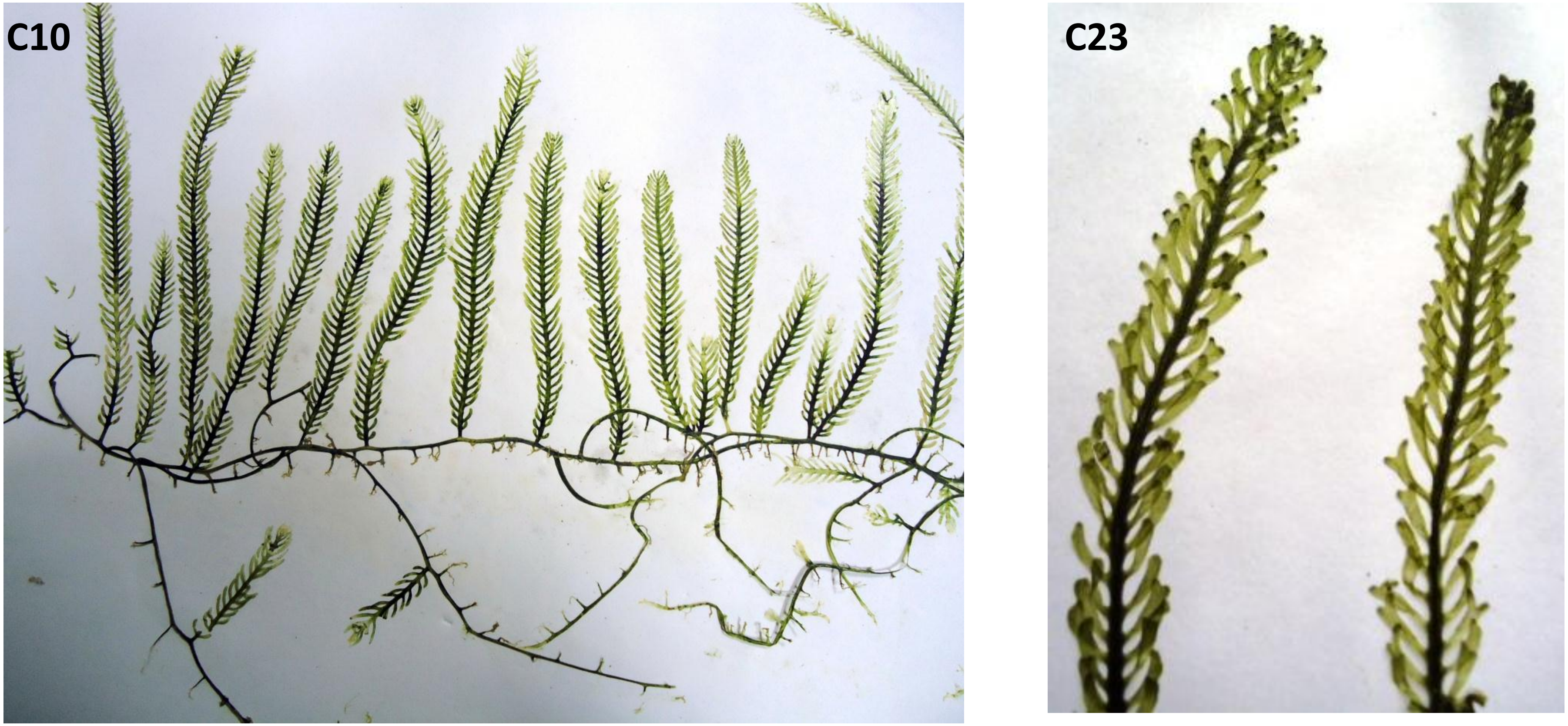

Description: Assimilators upto 21 cm in length, 7-15 mm in width, occasionally forked, flat broad midrib pinnately divided; ramuli flat opposite to alternate, with round apex, 5-7 mm long & 1.0-1.5 mm wide, occasionally bifurcate.

Figure S9; Specimens C10, C23

Caulerpa racemosa **(Forsskål) J. Agardh var.** *occidentalis* **(J. Agardh) Børgesen**

References: [3]: figs. 5 & 5a. [14]: p. 44. [44]: pl. 6, figs. 53-57. [46] pl. 17, fig. 6; pl. 18, fig. 5. [69]: figs. 5 & 6.

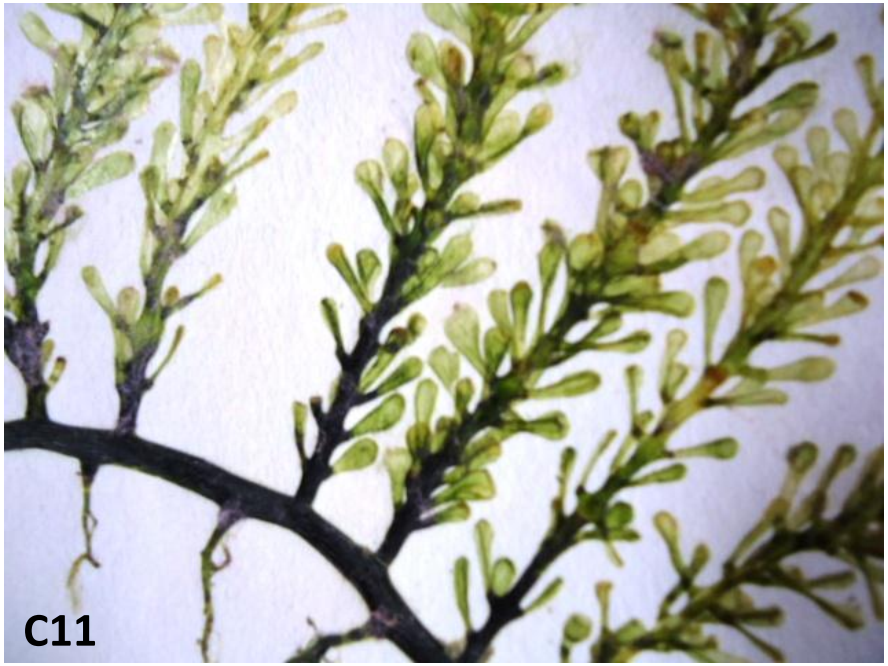

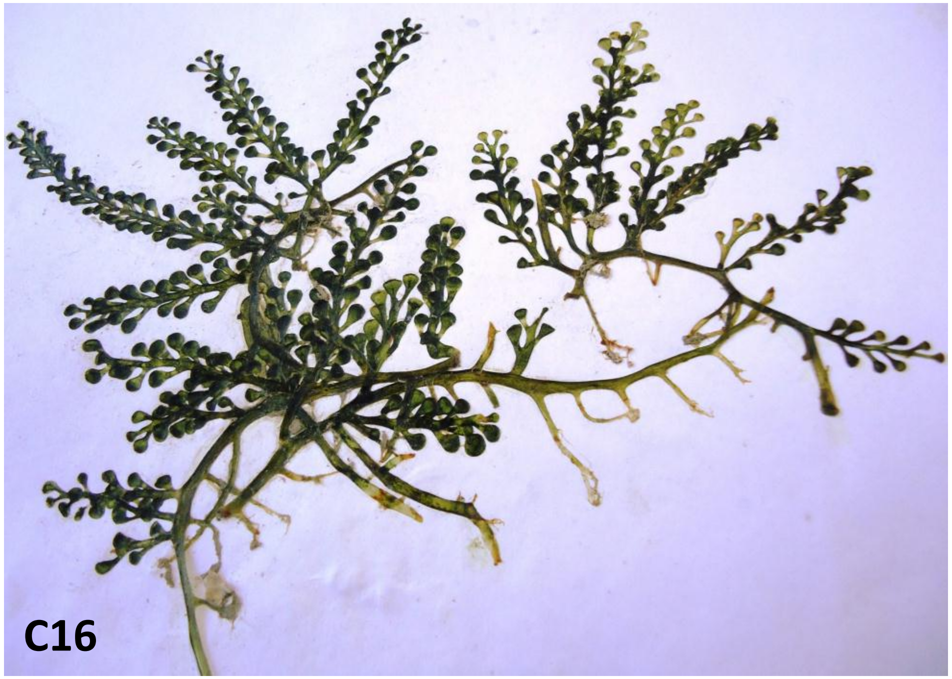

Description: Erect assimilators with branchlets not crowded, branchlets pear shaped, expanded to sub spherical top upto 2 mm diameter.

Figure S10; Specimens C11, C16

Caulerpa lentillifera **J. Agardh**

References: [6]: pl. XXXIV, figs. 1a, b. [47]: fig. 39-41. [51]: figs. 1-13. [66]: fig. 82. [69]: figs. 1-3.

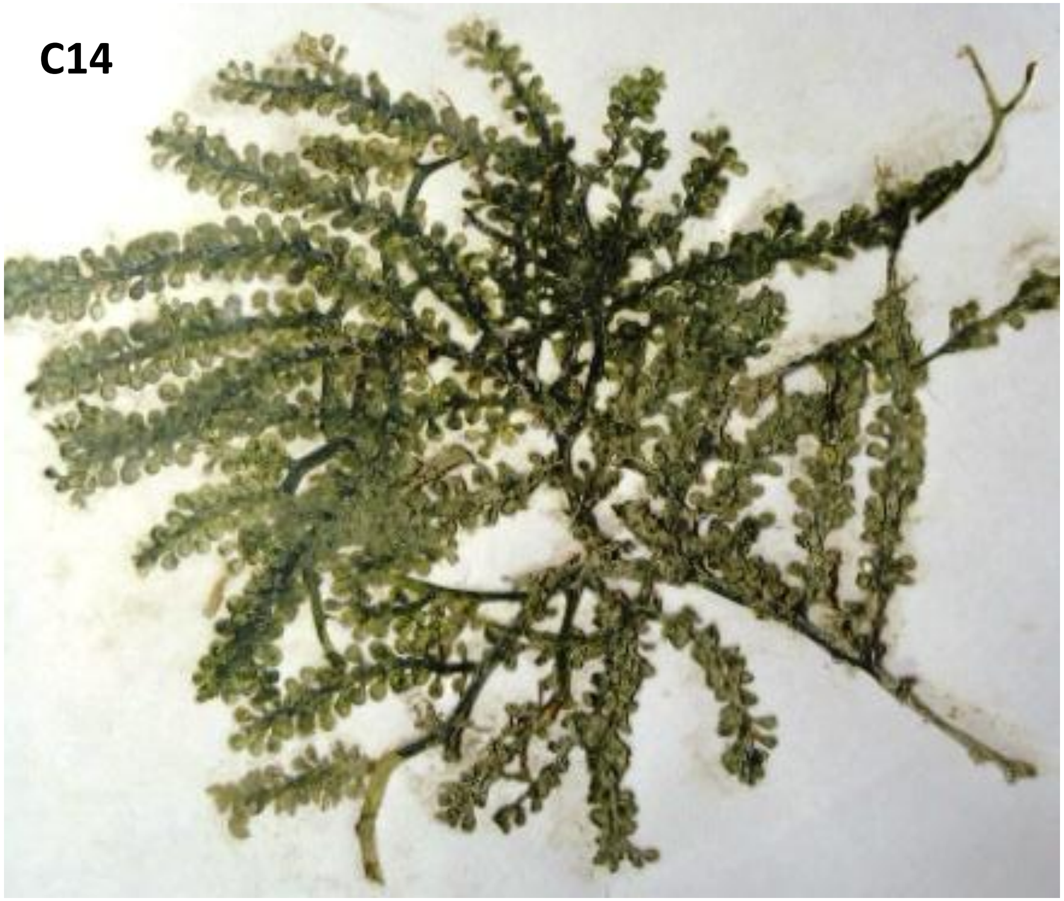

Description: Dark green colour, cylindrical stolon, branched rhizoids; erect assimilators upto 10 cm long densely covered with spherical to sub spherical ramuli; ramuli with constricted pedicel.

Figure S11; Specimen C14

Caulerpa racemosa **(Forsskål) J. Agardh var.** ***turbinata*** **(J. Agardh) Eubank**

References: [14]: p. 44. [44]: pl. 6, figs. 58 & 59. [52]: fig. 9 E. [69]: figs. 24-26.

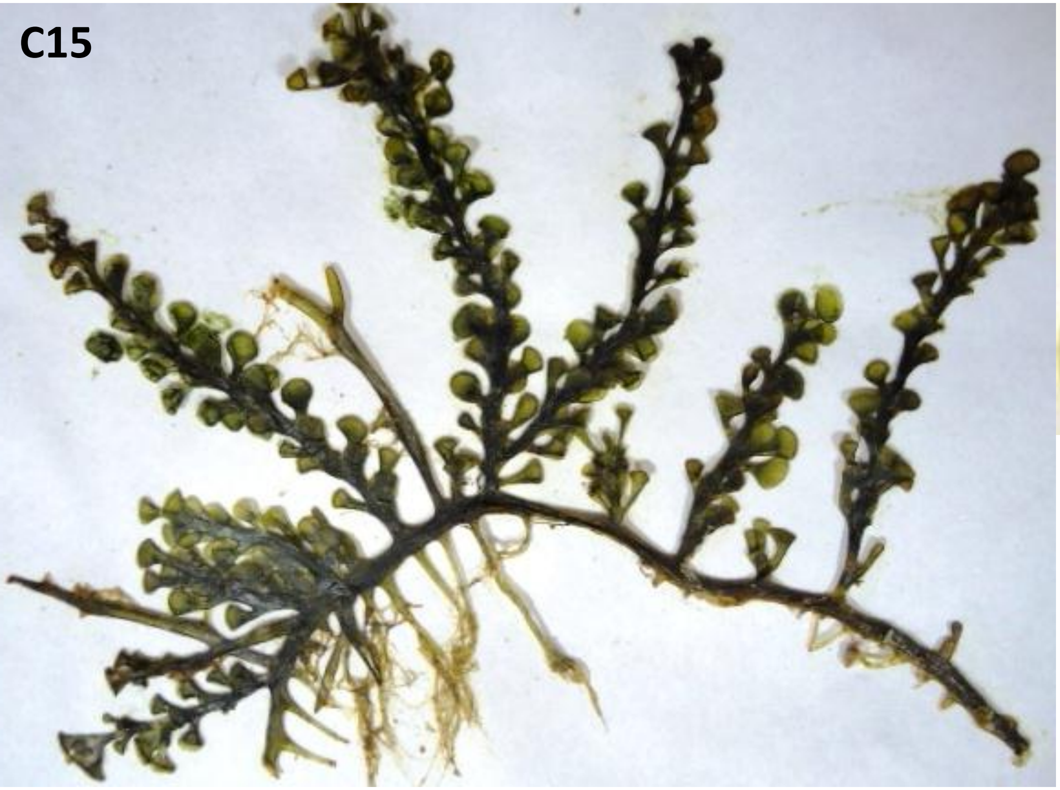

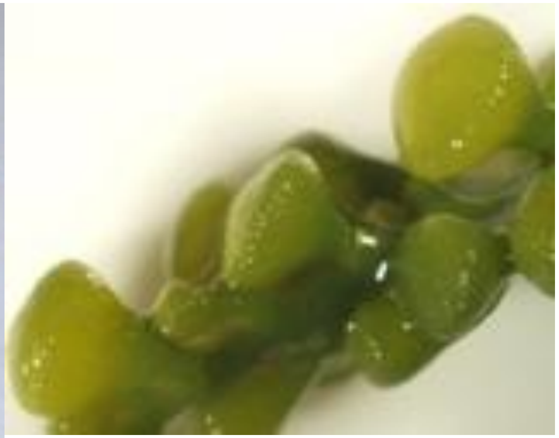

Description: Short erect assimilator with turbinate or trumpet or obconical shaped ramuli

Figure S12; Specimen C15

Caulerpa peltata **Lamouroux**

References : [3] pl. 3, [13]: fig. 2, A-I & 10 D. [14]: p. 40. fig. 6. [44]: pl. 6, figs. 50-52; pl. 12, fig. 93. [46]: pl. 17, fig. 2; pl. 18, fig. 1. [52] fig. 8 A &10 A. [65]: fig. 5.

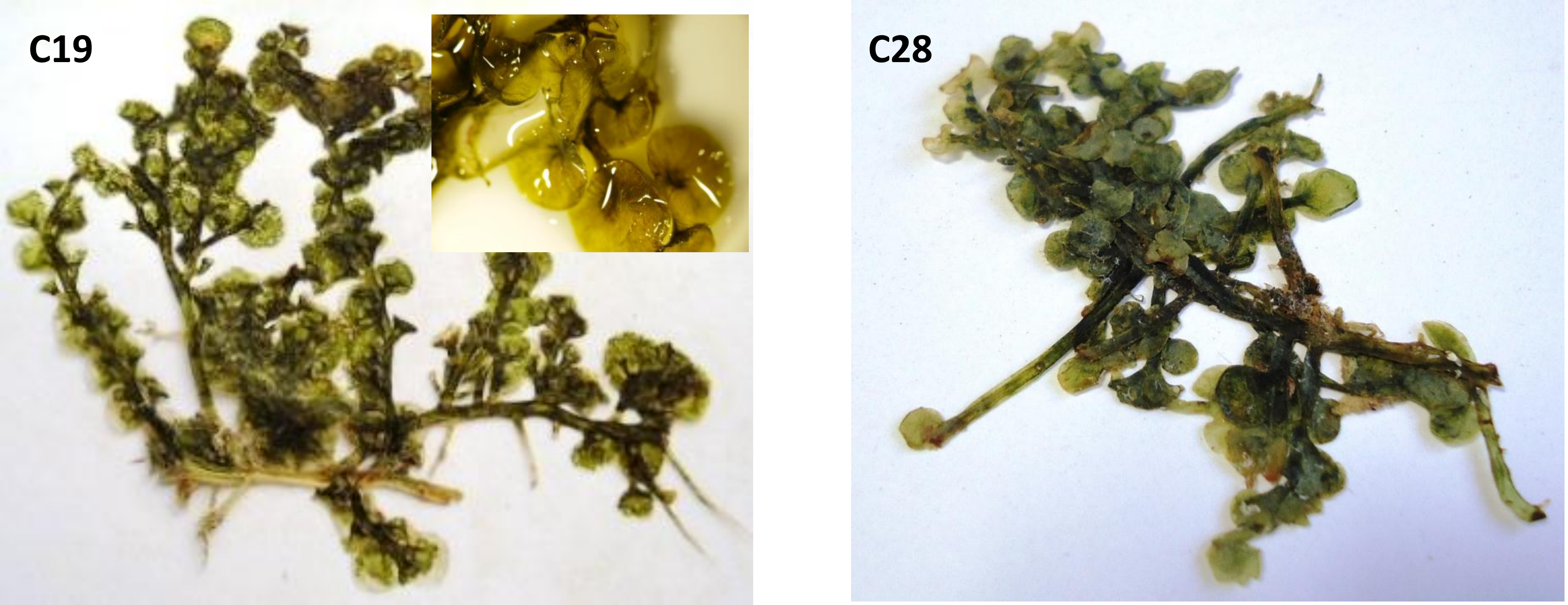

Description : Moderate size, creeping; short erect assimilators upto 4 cm long; ramuli distinctly peltate, radially arranged, ramuli disc 3-7 mm in diameter

Figure S13; Specimens C19, C28

Caulerpa serrulata **(Forsskål) J. Agardh**

References: [12] fig. 11, A-G. [43]: pl. XLVII. [46]: pl. 14, fig. 5. [48] figs. 24-25. [52]: 13A, B. [66]: fig. 91.

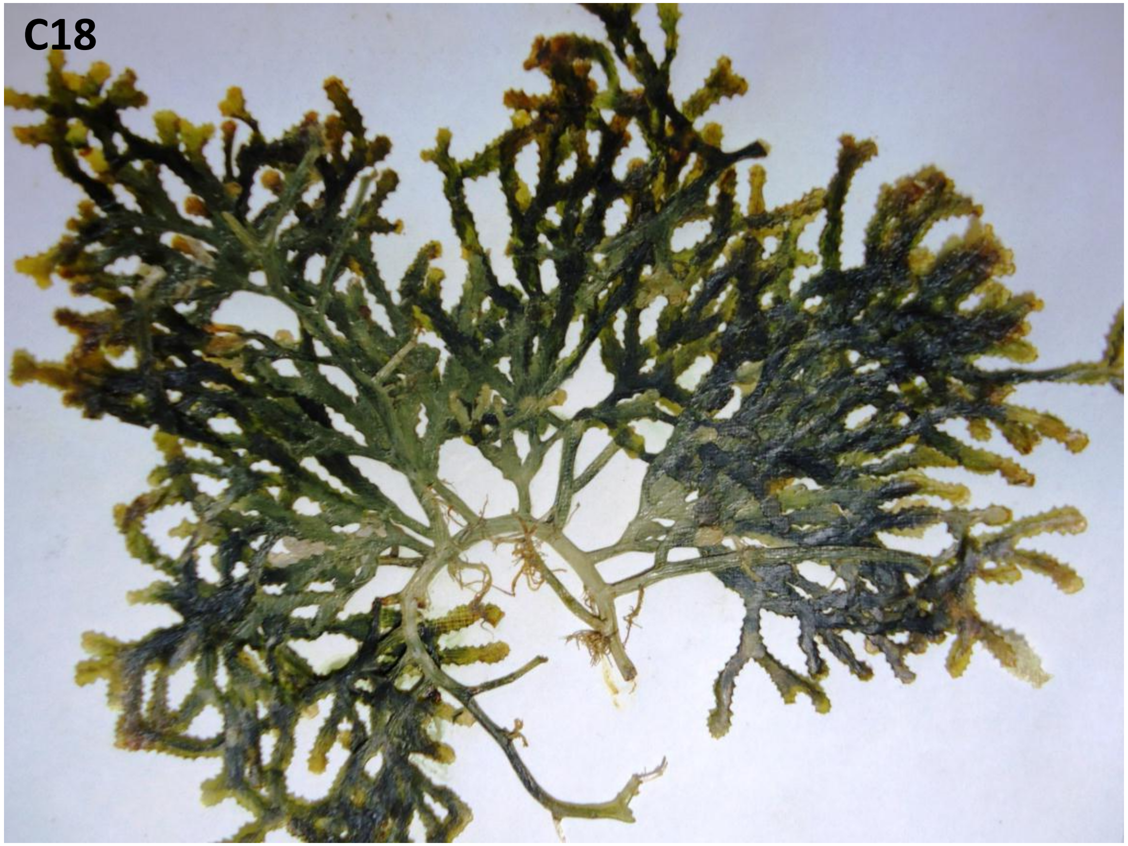

Description: Thick horizontal axis, assimilators strap-like and repeatedly branched, 2-6 cm long with dentate or serrated margins

Figure S14; Specimen C18

Caulerpa racemosa **(Forsskål) J. Agardh var.** *laetevirens* **(Mont.) Weber Bosse**

References: [3]: pl. IIa, figs. 4 & 4a as *C. laetevirens*. [6]: pl. XXXIII, figs. 16a, b, c. [13]: figs. 5, I-O & 10 F as *C. laetevirens*. [52]: fig 10C. [53]: 693, fig. 16 A–C as *C. racemosa* ecad *laetevirens*. [69]: figs. 21–23.

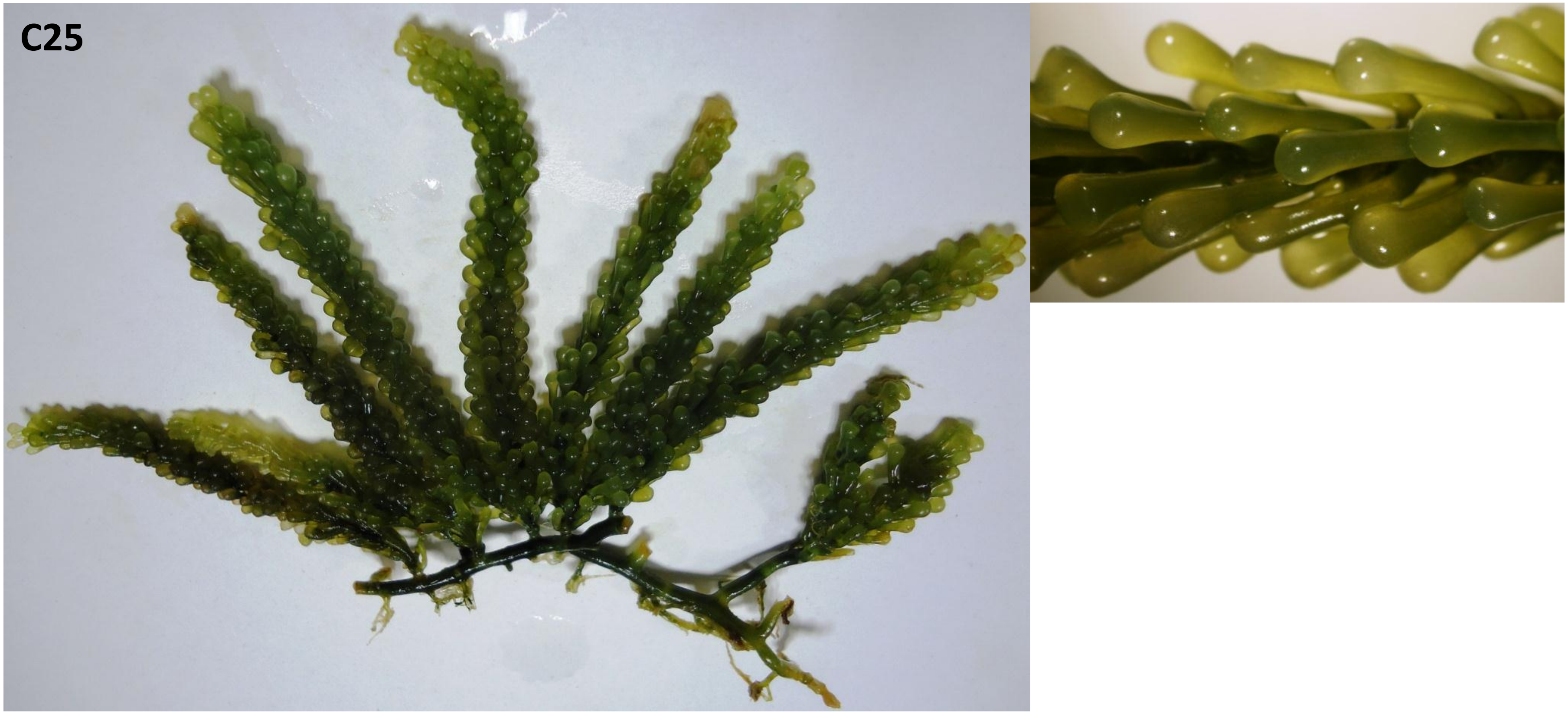

Description: Branched rhizoids; erect assimilators, cylindrical upto 4 cm long; radially arranged ramuli, cylindrical with swollen apices.

Figure S15; Specimen C25

Caulerpa mexicana **Sonder ex Kützing**

References: [12]: fig. 6, A-I. [46]: pl. 12, figs. 2-5. [48]: figs. 14-18, 34 & 35. [54]: figs. 37 & 38.

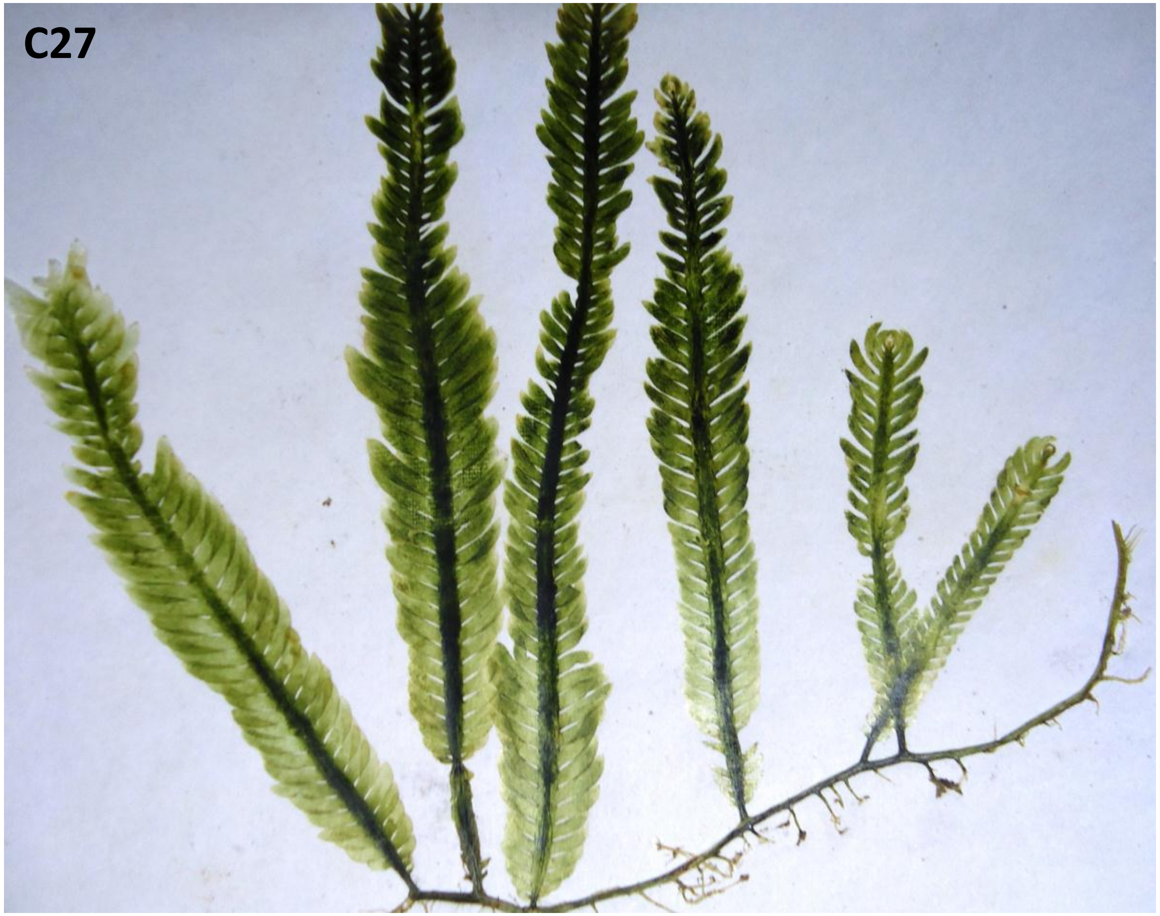

Description: Stoloniferous plant with branched rhizoid descending from rhizome at interval of few millimeters, fronds high upto 11 cm, 15 mm broad; pinnae opposite, flattened, acuminate not contracted at base.

Figure S16; Specimen C27

***Caulerpa racemosa* (Forsskål) J. Agardh var. *racemosa* f. *remota* (Svedelius) Coppejans comb. nov.**

References: [66]: fig. 89.

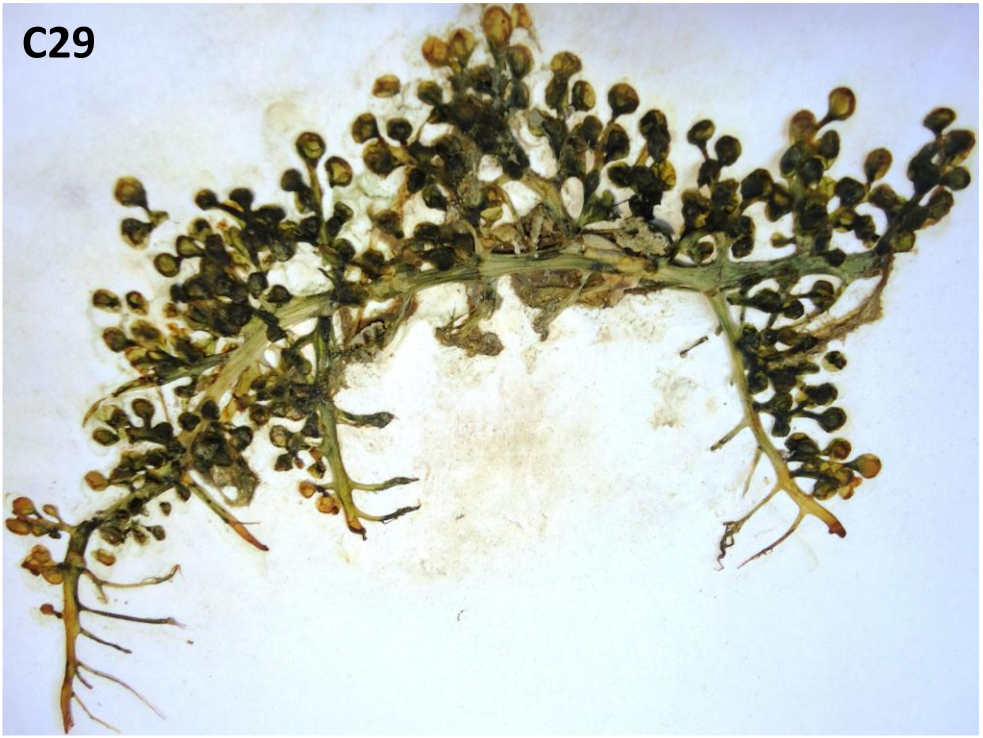

Description: Specimens very well agree with the description by Coppejans et al. (2009) except the length of rachis ranges from 1.5 – 5 cm. Thick rhizome with well separated assimilators; ramuli upto 4 mm attached to the assimilators with longer pedicels of length upto 3mm.

Figure S17; Specimen C29

Caulerpa **sp. C03**

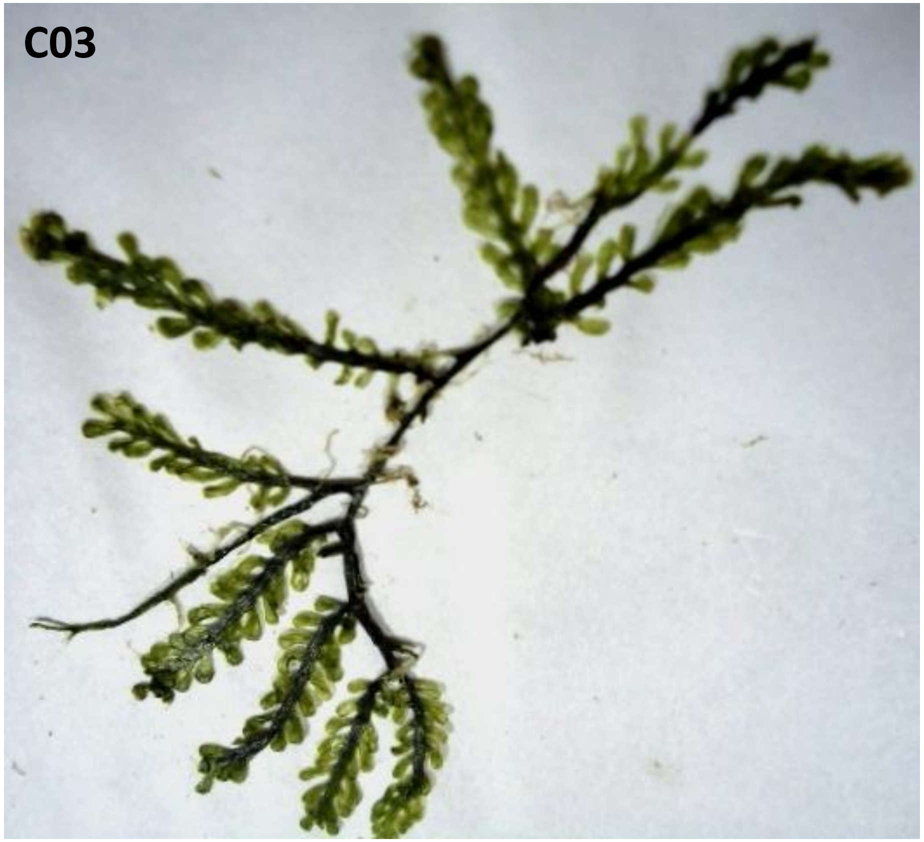

Description: Small size plant; erect assimilators upto 3.5 cm long; ramuli flat, incurved, slightly swelled towards top, opposite, distichously arranged on assimilator axis.

Figure S18; Specimen C03

***Caulerpa* sp. C13**

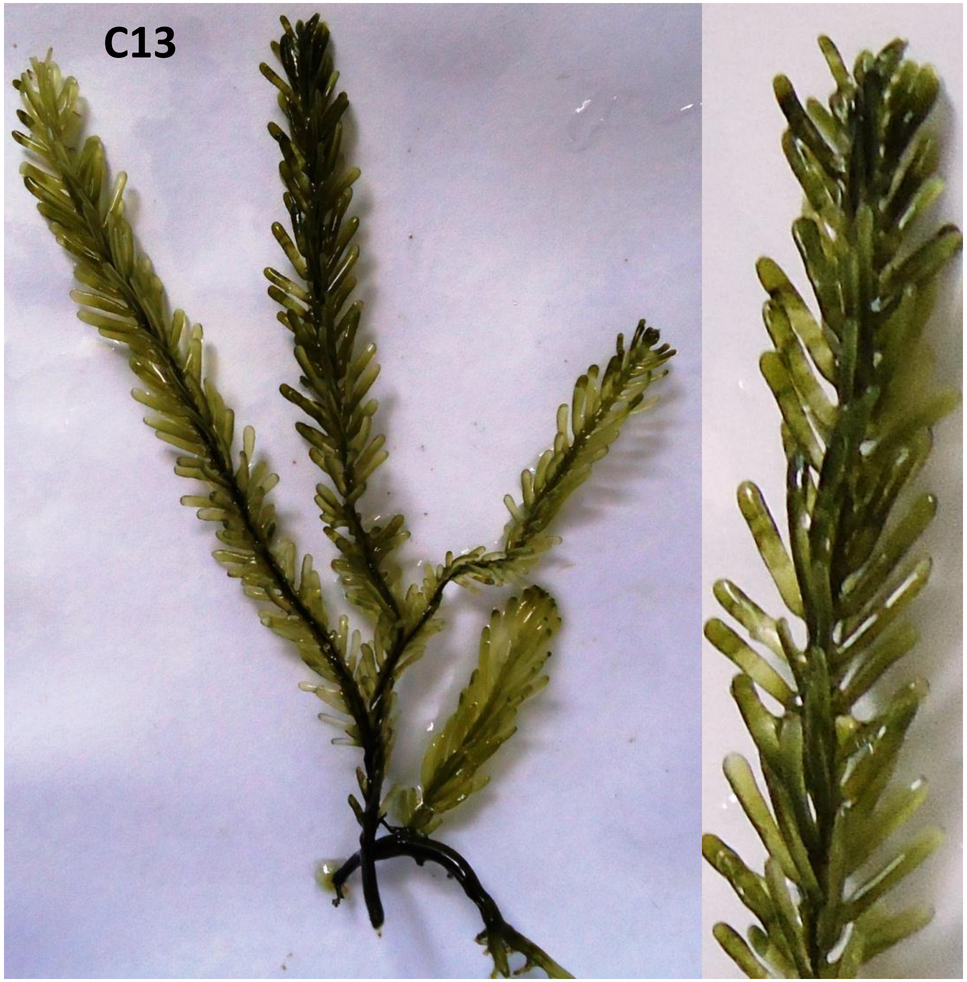

Description: Cylindrical rhizome; erect assimilator, occasionally branched, upto 7 cm tall; ramuli flattened or cylindrical, with rounded apex, radially arranged on central axis completely hiding the rachis.

Figure S19; Specimen C13
