# Supplementary figures and images for: Molecular Phylogeny and Barcoding of Caulerpa (Bryopsidales) Based on the tufA, rbcL, 18S rDNA and ITS rDNA Genes
Source: PLoS One. 2013 Dec 5;8(12):e82438. doi: 10.1371/journal.pone.0082438 (PMC3855484; doi:10.1371/journal.pone.0082438)

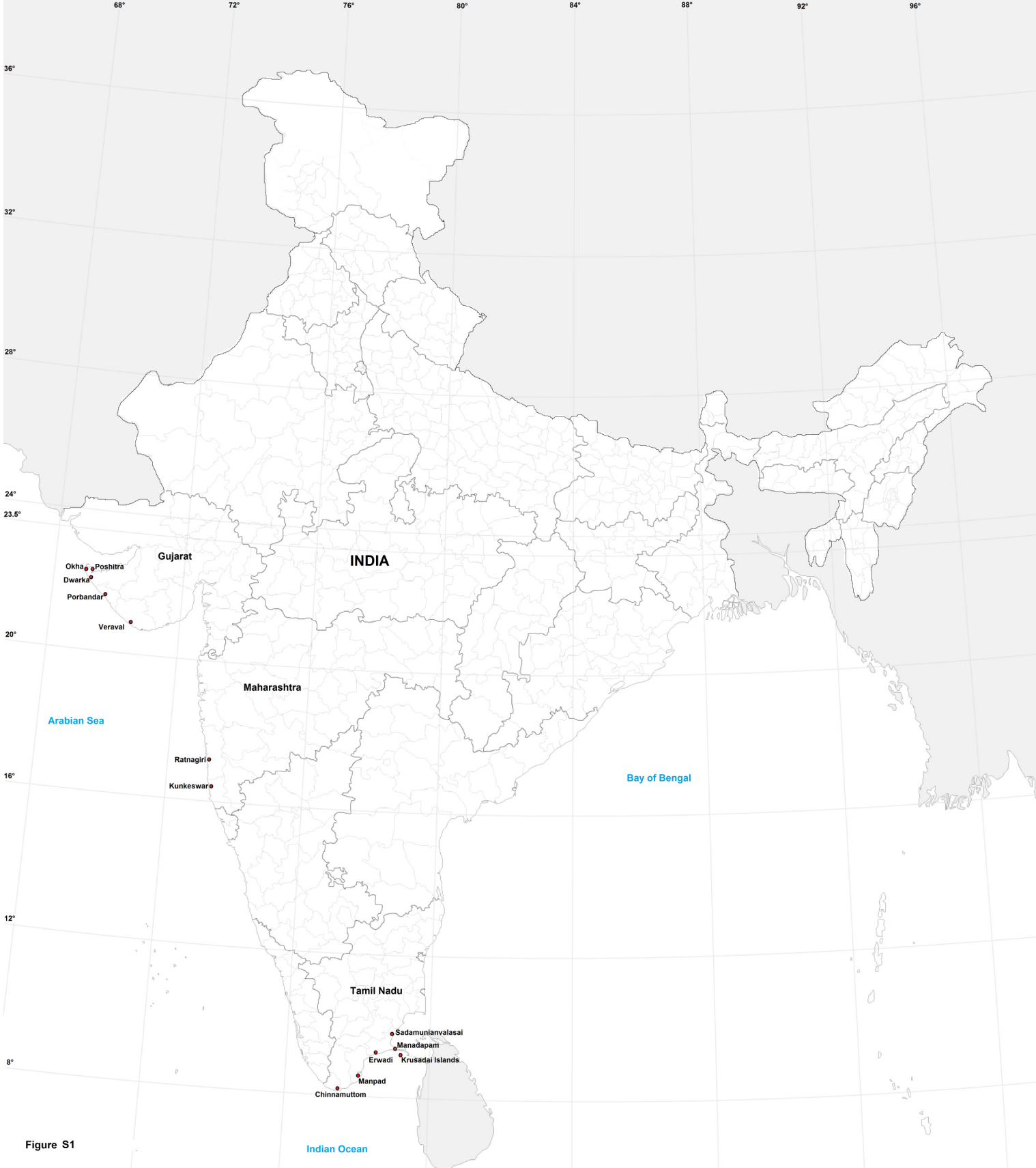

Figure S1

Supplement: Figure S1 — Sample collection sites from India. (PDF) [file pone.0082438.s002.pdf]

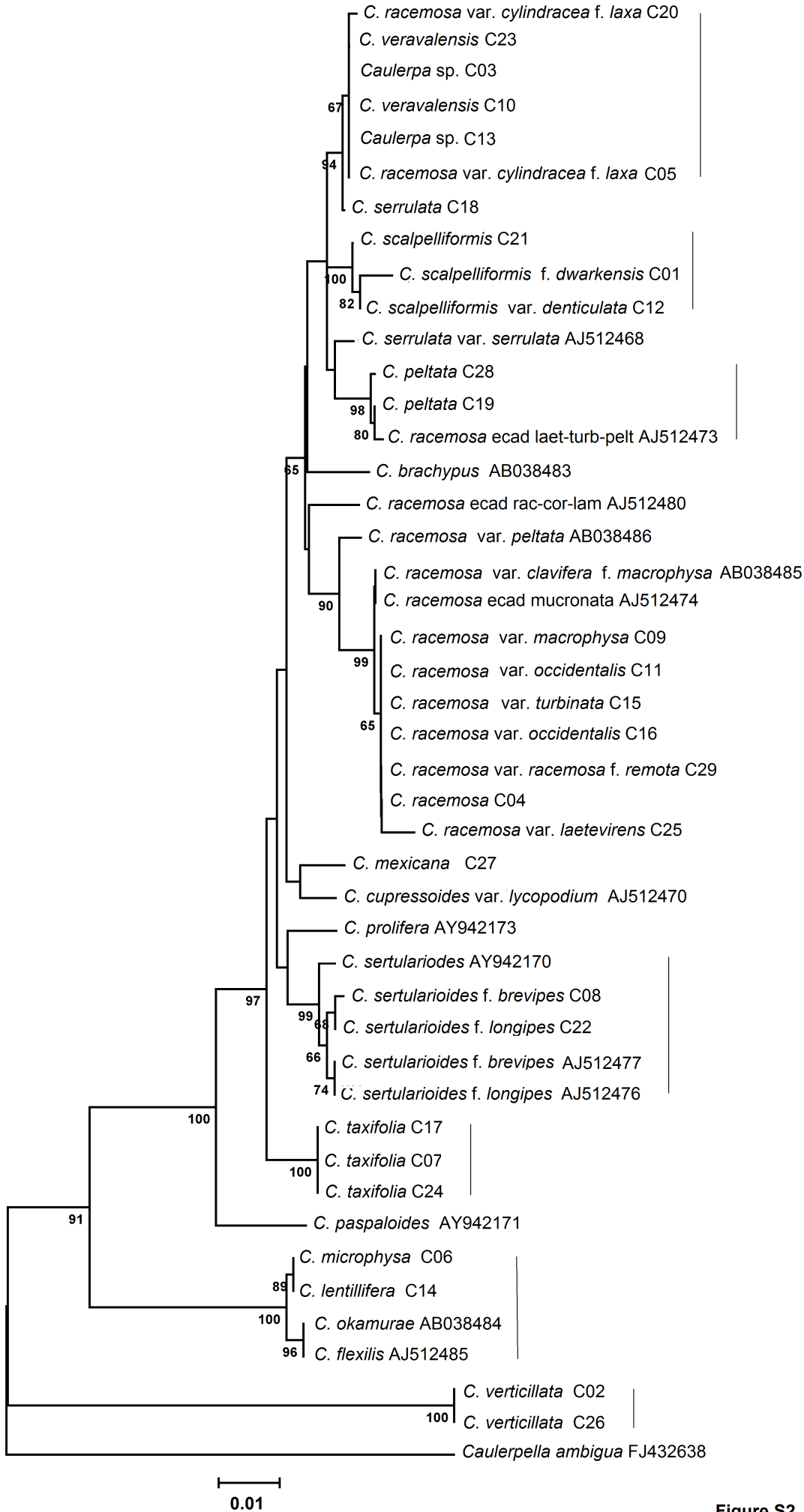

Figure S2

Supplement: Figure S2 — NJ tree based on rbcL gene sequence data. Support values at nodes correspond to bootstrap proportion (BS). Sample ID for specimens from this study and accession numbers for the reference sequences are given for identification in Table S1. Solid lines on the right indicate possible clades. (PDF) [file pone.0082438.s003.pdf]

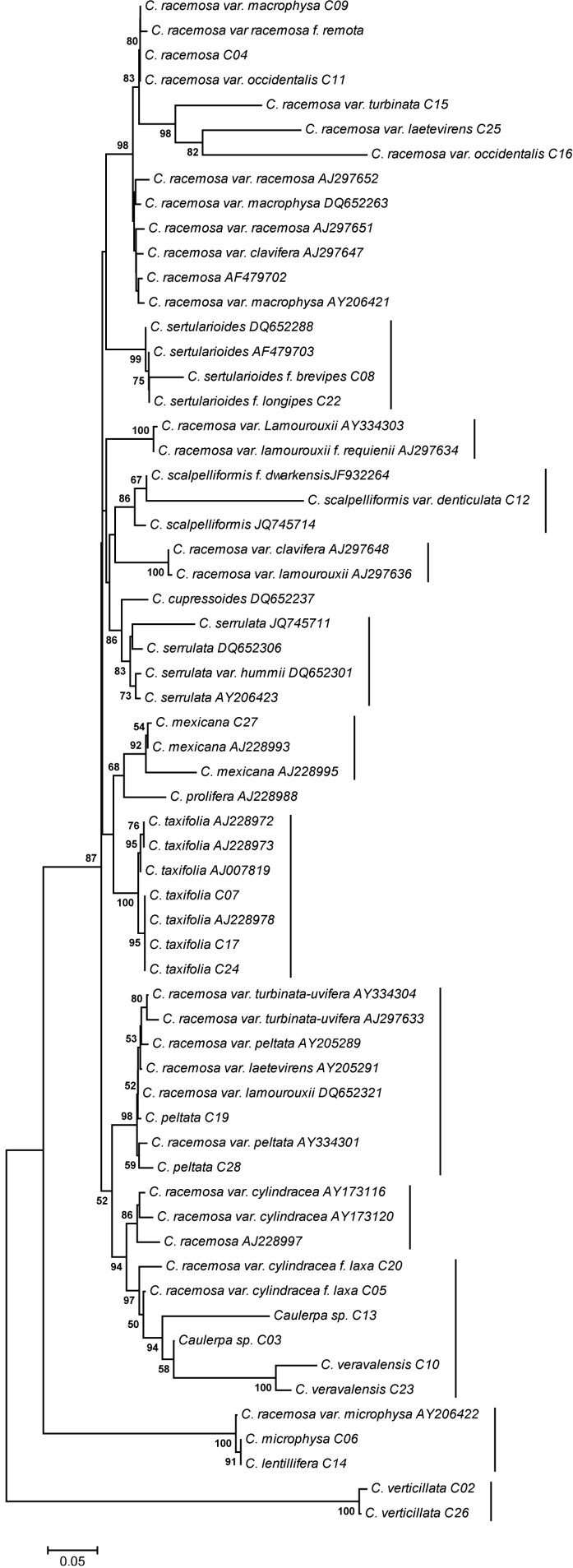

Figure S3

Supplement: Figure S3 — NJ tree based on ITS rDNA gene sequence data. Support values at nodes correspond to bootstrap proportion (BS). Sample ID for specimens from this study and accession numbers for the reference sequences are given for identification in Table S1. Solid lines on the right indicate possible clades. (PDF) [file pone.0082438.s004.pdf]
