# Supplementary material for: Molecular Phylogeny and Barcoding of Caulerpa (Bryopsidales) Based on the tufA, rbcL, 18S rDNA and ITS rDNA Genes
Source: PLoS One. 2013 Dec 5;8(12):e82438. doi: 10.1371/journal.pone.0082438 (PMC3855484; doi:10.1371/journal.pone.0082438)
Supplement: Table S1 — Specimens used in analyses, specimen voucher number, collection locations, collection date and accession numbers. (PDF) [file pone.0082438.s005.pdf]

Table S1. Specimens used in analyses, specimen voucher number, collection locations, collection date and accession numbers.

ID	Taxon name	Specimen #	Collection location	Collection date	Source of data	Accession no.			
						18S	*rbc*L	ITS	*tuf*A
C01	*Caulerpa scalpelliformis* (Brown ex Turner) C. Agardh f. dwarkensis Børgesen	19102009001	Veraval, Gujarat, India N 20º 54.61'; E 70º 20.81'	19-10-2009	This study	JF932251	JF932277	JF932264	KC153492
C02	*C. verticillata* J. Agardh	17102009001	Okha, Gujarat, India N 22º 28.68'; E 69º 4.23'	17-10-2009	This study	JF932252	JF932278	JF932265	KC153493
C03	*Caulerpa* sp.	3122009001	Porbandar, Gujarat, India N21º 38.05'; E 69º 36.24'	03-12-2009	This study		JF932279	JF932266	KC153494
C04	*C. racemosa* (Forsskål) J. Agardh	4122009001	Veraval, Gujarat, India N 20º 54.73'; E 70º 20.69'	04-12-2009	This study	JF932254	JF932280	JF932267	KC153495
C05	*C. racemosa* (Forsskål) J. Agardh var. cylindracea (Sonder) Verlaque, Huisman & Boudouresque f. laxa (Greville) Weber-van Bosse	3122009002	Porbandar, Gujarat, India N21º 38.05'; E 69º 36.24'	03-12-2009	This study	JF932255	JF932281	JF932268	KC153496
C06	*C. microphysa* (Weber van Bosse) J. Feldmann	2122009001	Okha, Gujarat, India N 22º 28.68'; E 69º 4.23'	02-12-2009	This study	JF932256	JF932282	JF932269	KC153497
C07	*C. taxifolia* (Vahl) C. Agardh	2122009002	Okha, Gujarat, India N 22º 28.68'; E 69º 4.232'	02-12-2009	This study	JF932257	JF932283	JF932270	KC153498
C08	*C. sertularioides* (S. Gmelin) Howe f. brevipes (J. Agardh) Svedelius	3122009003	Porbandar, Gujarat, India N21º 38.05'; E 69º 36.24'	03-12-2009	This study	JF932258	JF932284	JF932271	KC153499
C09	*C. racemosa* (Forsskål) J. Agardh var. macrophysa (Sonder ex Kützing) Taylor	29042010001	Okha, Gujarat, India N 22º 28.54'; E 69º 4.46'	29-04-2010	This study	JF932259	JF932285	JF932272	KC153500
C10	*C. veravalensis* Thivy & Chauhan	2112010001	Veraval, Gujarat, India N 20º 54.81'; E 70º 20.56'	02-11-2010	This study	JF932260	JF932286	JF932273	KC153501
C11	*C. racemosa* (Forsskål) J. Agardh var. occidentalis (J. Agardh) Børgesen	2122010003	Okha, Gujarat, India N 22º 28.82'; E 69º 4.68'	02-12-2010	This study	JF932261	JF932287	JF932274	KC153502
C12	*C. scalpelliformis* (Brown ex Turner) C. Agardh var. *denticulata* Børgesen	2122010002	Okha, Gujarat, India N 22º 28.82'; E 69º 4.68'	02-12-2010	This study	JF932262	JF932288	JF932275	KC153503
C13	*Caulerpa* sp.	2122010001	Okha, Gujarat, India N 22º 28.82'; E 69º 4.68'	02-12-2010	This study		JF932289	JF932276	KC153504
C14	*C. lentillifera* J. Agardh	20110419014	Poshitra rocks, Gujarat, India N 22º 24.34'; E 69º 12.19'	20-04-2011	This study	JN034412	JN034416	JN034414	KC153505

C15	*C. racemosa* (Forsskål) J. Agardh var. turbinata (J. Agardh) Eubank	29042010002	Okha, Gujarat, India N 22º 28.54'; E 69º 4.46'	29-04-2010	This study	JN034413	JN034417	JN034415	KC153506
C16	*C. racemosa* (Forsskål) J. Agardh var. occidentalis (J. Agardh) Børgesen	20110420003	Poshitra rocks, Gujarat, India N 22º 24.34'; E 69º 12.19'	20-04-2011	This study	JQ745681	JQ745695	JQ745709	KC153507
C17	*C. taxifolia* (Vahl) C. Agardh	20110420001	Poshitra rocks, Gujarat, India N 22º 24.34'; E 69º 12.19'	20-04-2011	This study	JQ745682	JQ745696	JQ745710	KC153508
C18	*C. serrulata* (Forsskål) J. Agardh	20101230001	Sadamunian, Tamilnadu, India N 09º 11.25'; E 78º 43.5'	30-12-2010	This study	JQ745683	JQ745697	JQ745711	KC153509
C19	*C. peltata* Lamouroux	20110103001	Manapadu, Tamilnadu, India N 08º 22.31'; E 78º 3.35'	03-01-2011	This study	JQ745684	JQ745698	JQ745712	KC153510
C20	*C. racemosa* (Forsskål) J. Agardh var. cylindracea (Sonder) Verlaque, Huisman & Boudouresque f. laxa (Greville) Weber-van Bosse	20110106001	Chinnamuttam, Tamilnadu, India N 08º 5.33'; E 77º 33.50'	06-01-2011	This study	JQ745685	JQ745699	JQ745713	KC153511
C21	*C. scalpelliformis* (Brown ex Turner) C. Agardh	20110104001	Mandapam, Tamilnadu, India N 09º 12.28'; E 78º 43.34'	04-01-2011	This study	JQ745686	JQ745700	JQ745714	KC153512
C22	*C. sertularioides* (S. Gmelin) Howe f. *longipes (J. Agardh) Collins*	20110419019	Poshitra rocks, Gujarat, India N 22º 24.34'; E 69º 12.19'	19-04-2011	This study	JQ754687	JQ745701	JQ745715	KC153513
C23	*C. veravalensis* Thivy & Chauhan	20101202001	Okha, Gujarat, India N 22º 28.82'; E 69º 4.68'	02-12-2010	This study	JQ745688	JQ745702	JQ745716	KC153514
C24	*C. taxifolia* (Vahl) C. Agardh	20110223001	Kunkeshwar, Maharashtra, India N 16º 20.2'; E 73º 23.24'	23-02-2011	This study	JQ745689	JQ745703	JQ745717	KC153515
C25	*C. racemosa* (Forsskål) J. Agardh var. *laetevirens (Mont.) Weber Bosse*	645	Okha, Gujarat, India N 22º 28.74'; E 69º 4.59'	24-11-2011	This study	JQ745690	JQ745704	JQ745718	KC153516
C26	*C. verticillata* J. Agardh	20110110001	Krusadai Island, Tamilnadu, India N 09 º 15.3'; E 79º 12'	10-01-2011	This study	JQ745691	JQ745705	JQ745719	KC153517
C27	*C. mexicana* Sonder ex Kützing	20101230002	Erwadi, Tamilnadu, India N 09º 12.28'; E 78º 43.34'	30-12-2010	This study	JQ745692	JQ745706	JQ745720	KC153518
C28	*C. peltata* Lamouroux	20110225001	Ratnagiri, Maharashtra, India N 17º 1.27'; E 73º 16.28'	25-02-2011	This study	JQ745693	JQ745707	JQ745721	KC153519

C29	*C. racemosa* (Forsskål) J. Agardh var. racemosa f. remota (Svedelius) Coppejans comb. nov.	20101230003	Erwadi, Tamilnadu, India N 09º 12.28'; E 78º 43.34'	30-12-2010	This study	JQ745694	JQ745708	JQ745722	KC153520
	C. taxifolia		Enoshima aquarium, Japan		[20]			AJ228972	
	C. taxifolia		Oahu aquarium, Hawaii, U.S.A.		[20]			AJ228973	
	C. taxifolia		Guadeloupe island, Caribbean		[20]			AJ228978	
	C. mexicana		Martinique I., Caribbean		[20]			AJ228993	
	C. mexicana		Stot-Yam, Israel, Mediterranean		[20]			AJ228995	
	C. racemosa		Marseille,Provence, France		[20]			AJ228997	
	C. prolifera		Martinique I., Caribbean		[20]			AJ228988	
	C. racemosa	98-197	Portobelo, Panama (Atlantic)		[40]	AF479702			
	C. sertularioides	98-190	Portobelo, Panama (Atlantic)		[40]	AF479703			
	C. racemosa var. *peltata*	YW01070601	Dali, Taiwan		[26]			AY205289	
	C. racemosa var. microphysa	YW01042203	Green Island, Taiwan		[26]			AY206422	
	C. serrulata	YW01042204	Green Island, Taiwan		[26]			AY206423	
	C. racemosa var. *laetevirens*	YW00050501	Kenting, Taiwan		[26]			AY205291	
	C. racemosa var. *macrophysa*	YW01031201	Nanwan, Taiwan		[26]			AY206421	
	C. racemosa var. *turbinata-uvifera*		Salakta, Tunisia		[24]			AJ297633	
	C. racemosa var. *lamourouxii* f. *requienii*		Cyprus		[24]			AJ297634	
	C. racemosa var. *lamourouxii*		Ishigaki, Japan		[24]			AJ297636	
	C. racemosa var. *clavifera*		Indian Ocean		[24]			AJ297647	
	C. racemosa var. *clavifera*		Ishigaki, Japan		[24]			AJ297648	
	C. racemosa var. *racemosa*		Heron I., Queensland, Australia		[24]			AJ297651	
	C. racemosa var. racemosa		Balicasag I., Philippines		[24]			AJ297652	
	C. taxifolia	RH9801811	Between Ganzirri andTorre Faro, Strait of Messina, Sicily		[21]			AJ007819	
	C. serrulata var. *hummii*		Chino Hills, CA		[28]			DQ652301	
	C. racemosa var. *lamourouxii*		Angeles, CA		[28]			DQ652321	
	C. racemosa var. *macrophysa*		Redondo Beach, CA		[28]			DQ652263	
	C. sertularioides		Orange, CA		[28]			DQ652288	
	C. cupressoides		US Virgin Islands		[28]			DQ652237	

C. serrulata	Alhambra, IL, US	[28]		DQ652306	
C. racemosa var. *turbinata-uvifera*	Saint-Barthe´lemy,	[55]		AY334304	
C. racemosa var. *lamourouxii*	Tripoli, Lebanon	[55]		AY334303	
C. racemosa var. *peltata*	Madeira, Atlantic	[55]		AY334301	
C. racemosa var. *cylindracea*	Carnac Island, Western Australia	[55]		AY173116	
C. racemosa var. *cylindracea*	Canary Islands, Spain	[56]		AY173120	
C. prolifera (Forsskål) J.V. Lamouroux	Florida Keys, USA	[27]	AY942173		
C. paspaloides (Bory de Saint-Vincent) Greville	Florida Keys, USA	[27]	AY942171		
C. sertulariodes	Florida Keys, USA	[27]	AY942170		
C. brachypus Harvey	Tokushima Pref. : Uchizuma Beach	[38]	AB038483		
C. okamurae Weber-van Bosse	Kanagawa Pref. : Aburatsubo, Miura city	[38]	AB038484		
C. racemosa var. *clavifera* f. *macrophysa*	Okinawa Pref. : Uken, Gushigawa city	[38]	AB038485		
C. racemosa var. *peltata*	Yamaguchi Pref. : Tohwa-cho	[38]	AB038486		
C. cupressoides var. *lycopodium*	Cape Bolinao (Pangasinan, NW Luzon Island, Philippines)	[17]	AJ512470		AJ512414
C. flexilis	Jerbis Bay, Australia	[17]	AJ512485		AJ512426
C. racemosa var. *laetevirens*	Cape Bolinao	[17]	AJ512473		AJ512415
C. racemosa var. *mucronata*	Cape Bolinao	[17]	AJ512474		AJ512417
C. racemosa	Cape Bolinao	[17]	AJ512480		AJ512425
C. serrulata var. *serrulata*	Cape Bolinao	[17]	AJ512468		AJ512411
C. sertularioides f. *brevipes*	Cape Bolinao	[17]	AJ512477		AJ512420
C. sertularioides f. *longipes*	Cape Bolinao	[17]	AJ512476		AJ512419
C. cupressoides var. *lycopodium*	Uken, Japan	[23]			AJ417928
C. cupressoides	St. Barthélemy, Lesser Antilles	[23]			AJ417929
C. serrulata	Dahab, Egypt	[23]			AJ417931
C. cupressoides var. *flabellata*	Cayo Carenero, Bocas del Toro, Panamá	[23]			AJ417930
C. scalpelliformis	Cape Banks, Australia	[23]			AJ417971
C. taxifolia	Moreton Bay, Australia	[23]			AJ417936
C. distichophylla	Cottesloe, Australia	[23]			AJ417940
C. ashmeadii	Long Key, Florida, USA	[23]			AJ417941
C. prolifera	Bali	[23]			AJ417942

	C. scalpelliformis var. *denticulata*	Damour, Lebanon	[23]		AJ417972
	C. sertularioides	Martinique, Lesser Antilles	[23]		AJ417944
	C. racemosa var. *macrophysa*	Galeta, Panamá	[23]		AJ417947
	C. mexicana	Cuba	[23]		AJ417951
	C. racemosa var. *peltata*	Isla Naos, Panamá	[23]		AJ417949
	C. racemosa var. *turbinata*	Dahab, Egypt	[23]		AJ417957
	C. racemosa var. *occidentalis*	Livorno, Italy	[23]		AJ417955
	C. racemosa var. *lamourouxii*	Uken, Japan	[23]		AJ417954
	C. selago	Abu Dhiab, Egypt	[23]		AJ417973
	C. filiformis	Bronte Beach, Australia	[23]		AJ417964
	C. brachypus (revised *to C.biserrulata* by Stam et. al. (2006))	Cangaluyan, Pangasinan	[23]		AJ417934
	C. subserrata	Uken, Japan	[23]		AJ417935
	C. webbiana	Dahab, Egypt	[23]		AJ417958
	C. webbiana var. *pickeringii*	N. Kwa-Zulu Natal, South Africa	[23]		AJ417966
	C. lanuginosa	Content keys, Florida, USA	[23]		AJ417959
	C. paspaloides	Long Key, Florida, USA	[23]		AJ417965
	C. microphysa	Texas Flower Gardens, USA	[23]		AJ417961
	C. cactoides	Jervis Bay, Australia	[23]		AJ417969
	C. geminata	Coffs Harbour, Australia	[23]		AJ417968
	C. verticillata	Long Key, Florida, USA	[23]		AJ417967
	C. flexilis	Jervis Bay, Australia	[23]		AJ417970
	C. microphysa		[28]		DQ652514
	C. prolifera		[28]		DQ652375
	C. serrulata		[28]		DQ652352
	C. taxifolia		[28]		DQ652357
	C. ashmeadii		[28]		DQ652362
	C. cupressoides		[28]		DQ652335
	C. brachypus		[28]		DQ652353
	C. mexicana		[28]		DQ652433
	C. paspaloides		[28]		DQ652504
	C. lanuginosa		[28]		DQ652496
	C. racemosa var. *cylindracea*	France	[18]		JN645150
	C. racemosa var. *cylindracea*	South Australia	[18]		JQ894933
	C. integerrima	Eilat, Israel	[30]		FJ810424
	C. bartoniae	Western Cape Province, False Bay, South Africa	[30]		FJ810426
	C. urvilleana	Rose Atoll, Samoa	[18]		JN645172
Outgroups	*Caulerpella ambigua*	Texas Flower Gardens, USA	[23]		AJ417963
	Caulerpella ambigua		[29]	FJ432638	
